# Intermittent fasting promotes plaque regression and activates the BMAL1–MERTK efferocytosis axis

**DOI:** 10.1186/s12964-026-03140-3

**Published:** 2026-08-11

**Authors:** Zian Feng, Zhiheng Xia, Hui Zhang, Chong Fan, Zhenxiao Ma, Mengyao Wang, Ke Gong, Baofa Sun, Shuang Zhang, Yajun Duan, Buchun Zhang, Zequn Yin

**Affiliations:** 1 Department of Cardiology, Division of Life Sciences and Medicine, the First Affiliated Hospital of USTC, University of Science and Technology of China, Hefei, Anhui 230026 China; 2 Anhui Institute of Cardiovascular Disease, The First Affiliated Hospital of USTC, University of Science and Technology of China, Hefei, Anhui 230026 China; 3 Department of Anesthesiology, Division of Life Sciences and Medicine, The First Affiliated Hospital of USTC, University of Science and Technology of China, Hefei, Anhui 230026 China; 4 Department of Cardiology, Affiliated Anhui Provincial Hospital of Anhui Medical University, Anhui Medical University, Hefei, Anhui 230032 China; 5 College of Food and Biological Engineering, Hefei University of Technology, Hefei, Anhui 230601 China; 6 Department of Infectious Diseases, Nanfang Hospital, Southern Medical University, Guangzhou, 510515 China; 7 Department of Physiology, Faculty of Basic Medical Sciences, Hubei Key Laboratory of Embryonic Stem Cell Research, Hubei University of Medicine, Shiyan, Hubei 442000 China; 8 State Key Laboratory of Medicinal Chemical Biology, Frontiers Science Center for Cell Responses, College of Life Sciences, Nankai University, Tianjin, 300071 China

**Keywords:** Atherosclerosis, Efferocytosis, Intermittent fasting, Macrophage, Plaque regression, Systems genetics

## Abstract

**Background:**

Atherosclerotic cardiovascular disease can progress despite intensive lipid lowering, highlighting the need to identify local mechanisms that remodel established plaques. Intermittent fasting may engage metabolic, circadian, and immune pathways relevant to this process.

**Methods:**

We used genome-wide association study structural equation modeling to construct circulatory plaque burden (CPB) and integrated multi-omic data to prioritize candidate pathways. We then tested intermittent fasting in a baseline-controlled Ldlr⁻/⁻ mouse induction–intervention model and evaluated the BMAL1–MERTK efferocytosis axis in macrophages.

**Results:**

Systems-genetic analyses linked systemic atherosclerotic burden to nutrient-response, circadian, and efferocytosis-related pathways. In mice, intermittent fasting reduced plaque size relative to both the ad libitum-fed and week-14 Baseline groups and improved structural features of plaque stability without additional endpoint lipid lowering. Aortic and single-cell analyses prioritized macrophage BMAL1–MERTK signaling. BMAL1 activated the Mertk promoter and occupied the tested promoter region. Mertk re-expression rescued the efferocytosis defect caused by Bmal1 knockdown, whereas MERTK inhibition reversed the benefit of BMAL1 overexpression.

**Conclusions:**

In this mouse model, intermittent fasting promoted remodeling of established atherosclerotic plaques, with macrophage BMAL1–MERTK signaling and efferocytosis identified as contributing mechanisms. These findings provide mechanistic insight and highlight this lifestyle-linked axis as a candidate for future translational investigation alongside established lipid-lowering therapy.

**Supplementary Information:**

The online version contains supplementary material available at https://doi.org/10.1186/s12964-026-03140-3.

## Abstract (plain language summary)

Cardiovascular disease is often driven by atherosclerosis, in which fat, dead cells, and inflammatory debris accumulate within artery walls. Cholesterol-lowering medicines are effective but do not always stop established plaques from progressing. In mice, intermittent fasting reduced plaque size and improved features of stability despite endpoint blood lipid levels similar to those in freely fed controls. Macrophages act as "garbage collectors" by removing dead cells from plaques. Our experiments showed that BMAL1 increased MERTK expression and supported this clearance process; restoring Mertk rescued impaired clearance when Bmal1 was reduced. By integrating human genetics with animal and cell experiments, our study suggests that intermittent fasting may promote plaque remodeling partly through macrophage efferocytosis. These preclinical findings require further testing in humans.

**Supplementary Information:**

The online version contains supplementary material available at https://doi.org/10.1186/s12964-026-03140-3.

## Background

Atherosclerotic cardiovascular disease (ASCVD) remains a leading cause of morbidity and mortality worldwide. Intensive lipid lowering substantially reduces cardiovascular events, yet clinically significant residual risk persists after recommended lipid targets are achieved [1–3]. Serial imaging studies also show heterogeneous plaque responses, with regression occurring in some but not all patients despite very low LDL-C [4–7]. Residual inflammatory risk remains prognostically important during statin therapy [8]. These observations place the biology of established plaque remodeling alongside systemic lipid control as an important therapeutic question.

Intermittent fasting has emerged as a dietary approach [9–11] that may influence cardiovascular biology [12]. Its reported effects include changes in insulin sensitivity and adiposity [13, 14], oxidative-stress responses [15, 16], and inflammatory signaling [17]. These findings provide a rationale for studying fasting in atherosclerosis, but most work has focused on systemic risk factors or lesion development. Whether a defined fasting regimen can remodel already-established plaques, and which plaque-resident processes might mediate such an effect, remains less clear.

Intermittent-fasting protocols differ in fasting duration, timing, and feeding pattern. Mechanistic reviews and human studies have examined periodic fasting, early time-restricted feeding, and alternate-day schedules [18, 19]. Observational data on eating-window length have reported nonlinear associations with mortality that vary across population subgroups [20], while experimental work shows that metabolic and immune responses to fasting vary with host metabolic state [21]. These findings support precise description and context-specific interpretation of each fasting intervention [20].

Macrophage handling of dying cells is a central determinant of how established plaques evolve. Plaque macrophages contribute to lipid accumulation and inflammatory signaling [22], while also clearing apoptotic cells through efferocytosis [23]. Efficient clearance restrains secondary necrosis and supports tissue repair and plaque stability [24]. Mertk is a major macrophage receptor in this process, and impaired Mertk signaling promotes apoptotic-cell accumulation and plaque necrosis [25]. Bmal1, a core circadian regulator, also shapes myeloid inflammatory and metabolic programs [26], raising the possibility that recurring fasting-feeding cycles influence plaque remodeling through a Bmal1-Mertk connection.

Defective efferocytosis allows apoptotic cells to accumulate and undergo secondary necrosis, amplifying plaque inflammation [27, 28]. Several preclinical approaches support the broader principle that restoring macrophage clearance can favor inflammatory resolution. Interventions directed at IRF5 [29], SECTM1A-GITR-LXRα signaling [30], and other components of apoptotic-cell recognition and engulfment [31], including CD47-directed strategies [32, 33], have improved efferocytic activity in experimental settings. It remains unknown whether nutritional timing can engage an endogenous macrophage clearance program in established plaques and whether the same pathway is supported by human vascular data. Addressing this question requires evidence that connects population-level pathway prioritization with cell-resolved and experimental mechanism testing.

We therefore asked whether intermittent fasting could remodel established atherosclerotic plaques and whether macrophage Bmal1-Mertk signaling contributes to this response. We first integrated genetic information across multiple arterial phenotypes to prioritize nutrient-responsive and circadian pathways associated with systemic atherosclerotic burden [34]. We then combined an *Ldlr-/-*induction-intervention model with aortic transcriptomics, public human single-cell datasets, plaque histology, and macrophage experiments [35]. Our central hypothesis was that intermittent fasting enhances Bmal1-dependent Mertk expression and efferocytosis, thereby favoring remodeling of established plaques.

## Methods

### Reagents and antibodies

Dexamethasone (Cat# 40323ES25) was purchased from Yeasen Biotechnology (Shanghai, China). 4′,6-diamidino-2-phenylindole (DAPI) nuclear stain (Cat# D9542) and Oil Red O (Cat# O0625) were purchased from Sigma-Aldrich (St. Louis, MO, USA). A terminal deoxynucleotidyl transferase dUTP nick-end labeling (TUNEL) Apoptosis Assay Kit (Cat# A112-01) [36], Dual-Luciferase Reporter Assay Kit (Cat# DL101), HiScript II 1 st Strand cDNA Synthesis Kit (Cat# R211-01), and AceQ qPCR SYBR Green Master Mix (Cat# Q111-02) were purchased from Vazyme Biotech (Nanjing, China). The Hematoxylin–Eosin (HE) Stain Kit (Cat# G1120) and Elastica van Gieson (EVG) staining kit (Cat# G1597) were purchased from Solarbio (Beijing, China). CFDA-SE (Cat# C1031) was purchased from Beyotime Biotechnology (Shanghai, China), and CellTracker Red CMTPX (Cat# C34552), TRIzol (Cat# 15,596,026), Lipofectamine RNAiMAX (Cat# 13,778,075), and Lipofectamine 2000 (Cat# 11,668,019) were purchased from Thermo Fisher Scientific (Waltham, MA, USA).

Fetal bovine serum (FBS; Cat# S1303-500) was purchased from Wjcell Biotechnology (Shanghai, China). High-glucose Dulbecco's modified Eagle medium (DMEM; Cat# BC-M-005–500 ml) and Roswell Park Memorial Institute 1640 medium (RPMI-1640; Cat# BC-M-002–500 ml) were purchased from Bio-Channel (Nanjing, China), red blood cell lysis buffer (Cat# BL503B) from Biosharp (Hefei, China), recombinant murine macrophage colony-stimulating factor (M-CSF; Cat# 315–02) from PeproTech (Cranbury, NJ, USA), and oxidized low-density lipoprotein (ox-LDL; Cat# YB-002) from Yiyuan Biotechnologies (Guangzhou, China). Mouse Bmal1-targeting and negative-control small interfering RNAs (siRNAs) were synthesized by GenePharma (Shanghai, China). Primary antibodies included F4/80 (Cat# 28,463–1-AP), Bmal1 (Cat# 14,268–1-AP), Mac-2/Galectin-3 (Cat# 60,207–1-Ig), MMP9 (Cat# 60,600–1-Ig), CCL2/MCP-1 (Cat# 66,272–1-Ig), and IL-10 (Cat# 60,269–1-Ig), all from Proteintech (Wuhan, China); CD68 (Cat# YM3050) from ImmunoWay; and TNF-α (Cat# YP-mAb-15972) from UpingBio (China). A rabbit polyclonal Mertk antibody (Cat# ab95925; Abcam, Cambridge, UK) was used for single-marker plaque staining. The dual Mertk/FLT3 kinase inhibitor MRX-2843 (Cat# T16144; TargetMol) was used for pharmacological inhibition. Fluorescence-conjugated secondary antibodies against mouse and rabbit IgG (Cat# SA00013-1 and SA00013-2) were purchased from Proteintech.

### Animal models and dietary interventions

All animal experiments were approved by the Animal Ethics Committee of the Hefei Comprehensive National Science Center Institute of Health and Medicine (Approval No. IHM-AP-2024–025) and followed the NIH Guide for the Care and Use of Laboratory Animals. Eight-week-old male Ldlr⁻/⁻ mice on a C57BL/6 J background were purchased from GemPharmatech (Nanjing, China). Mice were housed in individually ventilated cages in a specific pathogen-free facility at 23 ± 1 °C and 60–70% relative humidity under a 12 h light/12 h dark cycle, with lights on at 08:00 and off at 20:00, and with food and water available ad libitum before the intervention. A total of 24 mice entered the study. Eight mice were assigned to each of the Baseline, ad libitum feeding, and intermittent fasting groups, and all animals completed the planned protocol without exclusion. The mouse was the independent experimental unit; biochemical and histological analyses included eight mice per group. The target group size was eight mice per group; no formal a priori power calculation was performed.

Mice were fed a proatherogenic diet containing 40 kcal% fat and 1.25% cholesterol (D12108C; Research Diets, New Brunswick, NJ, USA) for 14 weeks [37]. At week 14, all mice underwent blood collection for biochemical analysis, after which eight mice were selected using a computer-generated random-number sequence and euthanized as the Baseline group. The remaining 16 mice were switched to a normal diet and randomly allocated 1:1 to ad libitum feeding or 24 h intermittent fasting for 4 weeks using a computer-generated random-number sequence [38]. Intermittent fasting was defined as 24 h of complete fasting with water available followed by 24 h of ad libitum feeding, with this cycle repeated throughout the intervention. Food was removed at 20:00 and restored 24 h later at 20:00; thus, each fasting interval encompassed one complete light–dark cycle [12]. Body weight and food intake were recorded every 48 h. Terminal blood was collected from the ad libitum-fed and intermittent-fasting groups immediately before euthanasia. Serum was separated at 400 × g for 20 min at 4 °C and stored at − 20 °C. Total cholesterol, triglycerides, LDL-C, HDL-C, and nonesterified fatty acids were measured using a Hitachi 3100 automated biochemical analyzer.

### Histology and immunofluorescence analysis

The entire aorta, extending from the proximal ascending aorta through the aortic arch and thoracic and abdominal aorta to the iliac bifurcation, was isolated, and adventitial adipose tissue was removed under a stereomicroscope. The vessel was opened longitudinally, pinned flat, fixed in 4% paraformaldehyde for 24 h, and stained with 0.5% Oil Red O [39]. En face lesion burden was calculated as Oil Red O-positive area divided by total intimal surface area. Aortic roots were embedded in OCT and cut into serial 6-μm cryosections. Sections were retained beginning at the first appearance of the three aortic-valve leaflets and continuously collected through the three-leaflet region until this morphology was no longer present; approximately 15 sections were retained per mouse. For staining, sections were selected using randomized coded identifiers, with section numbers and anatomic levels matched as closely as possible across animals. Root sections were stained with Oil Red O, hematoxylin and eosin (HE), and Elastica van Gieson (EVG) [40]. Plaque area, necrotic-core area, fibrous-cap area, and collagen-related stability features were quantified from three anatomically level-matched sections per mouse. Section-level measurements were averaged within each mouse to yield one value for group-level analysis.

For immunofluorescence, sections were blocked in phosphate-buffered saline (PBS) containing 5% bovine serum albumin for 1 h and incubated overnight at 4 °C with the indicated primary antibodies. Mac-2 was used as a broad plaque macrophage marker. The prespecified co-staining combinations included Bmal1 with CD68 and the anti-inflammatory cytokine IL-10 with the macrophage marker F4/80; TNF-α, CCL2, and MMP9 were evaluated as marker-specific plaque signals. Mertk was assessed by single-marker staining, whereas TUNEL and Mac-2 co-staining was used to evaluate plaque apoptotic-cell burden, its spatial relationship to macrophages, and a histologic efferocytosis index. After washing, sections were incubated with the corresponding fluorescence-conjugated secondary antibodies for 1 h in the dark and counterstained with DAPI. TUNEL staining was performed according to the manufacturer's protocol [41]. For the MMP9 assay, parallel frozen sections were processed without the MMP9 primary antibody and then incubated with the corresponding fluorescent secondary antibody. Fluorescence persisting along the vascular elastic lamina in this primary-antibody-omission control was classified as arterial-wall autofluorescence rather than MMP9-specific signal [42, 43].

Three level-matched sections were analyzed for each mouse using a Zeiss LSM 900 confocal microscope. At least three nonoverlapping fields covering the plaque region were acquired per section under identical marker-specific settings. Thresholding, background subtraction, and region-of-interest definitions were held constant within each assay. Measurements from fields and sections were first averaged within each mouse, and each mouse contributed one value to statistical analysis. Mac-2-positive area and marker-specific signals were normalized to total plaque area as appropriate. Mertk was quantified from the single-marker images as relative mean fluorescence intensity (MFI) within the prespecified plaque region, calculated as integrated plaque-region fluorescence divided by plaque-region area, and was not normalized to a macrophage co-stain. MMP9 and CCL2 were quantified as relative mean fluorescence intensity (MFI) within the plaque region. Bmal1 was quantified as relative MFI within CD68-positive plaque regions. For IL10/F4/80 co-staining, IL10 served as an anti-inflammatory marker and F4/80 as a macrophage marker; the two fluorescence channels were quantified separately as relative MFI within the same prespecified plaque region of interest, and no separate co-localization metric was calculated. TUNEL-positive cell profiles within the plaque region were defined as apoptotic cells (ACs), and Mac-2 served as the spatial macrophage reference. An AC was classified as Mac-2-positive macrophage-associated when its red TUNEL signal overlapped with Mac-2 signal (yellow in merged images) or when the red AC was immediately contacted and encircled by green Mac-2 signal. TUNEL-positive ACs lacking adjacent or surrounding Mac-2 signal were classified as free ACs. The histologic efferocytosis index was calculated as the number of Mac-2-positive macrophage-associated ACs divided by the number of free ACs; a higher ratio indicates greater macrophage association relative to the accumulation of free apoptotic cells [24]. Elastic-lamina-adjacent fluorescence identified in the MMP9 primary-antibody-omission controls was excluded from the MMP9 measurement. Image quantification was performed in ImageJ using coded images. For all immunofluorescence analyses, regions of interest were restricted to the aortic-root valve plaque area. Signals from the outer arterial wall and elastic lamina were excluded because elastin-rich structures can produce nonspecific fluorescence and autofluorescence.

### Cell culture

Both the human Jurkat T-cell line and the HEK293T cell line were purchased from the American Type Culture Collection (ATCC; Manassas, VA, USA). Jurkat cells were cultured in suspension in standard Roswell Park Memorial Institute 1640 medium (RPMI-1640) supplemented with 10% fetal bovine serum (FBS) and 1% penicillin‒streptomycin. HEK293T cells were adherently cultured in DMEM (high glucose) supplemented with 10% FBS and 1% penicillin‒streptomycin. All the cell lines were maintained in a humidified incubator at 37 °C with 5% CO_2_, passaged regularly, and tested for mycoplasma contamination.

Primary bone marrow-derived macrophages (BMDMs) were isolated from the femurs and tibias of 4-week-old male C57BL/6 J wild-type mice. Marrow cells were flushed with ice-cold sterile PBS, centrifuged at 4 °C, and treated with red blood cell lysis buffer for 15 min on ice. Cells were washed and cultured in complete DMEM/F12 containing 10% FBS and 20 ng/mL M-CSF [44] for 7 days, with half-medium replacement every 2–3 days. The use of 4-week-old donors followed published BMDM protocols that generated mature macrophages after M-CSF differentiation, and all donor-specific cultures were matched for age, sex, and genetic background to limit preparation-related heterogeneity [45, 46].

### Macrophage intervention and in vitro efferocytosis assay

Differentiated BMDMs were exposed to 50 μg/mL ox-LDL for 24 h [38]. The loss-of-function/rescue experiment comprised four conditions: untreated control, ox-LDL alone, ox-LDL plus Bmal1 knockdown, and ox-LDL plus Bmal1 knockdown with Mertk re-expression. The complementary overexpression/inhibitor experiment comprised untreated control, ox-LDL alone, ox-LDL plus Bmal1 overexpression, and ox-LDL plus Bmal1 overexpression with MRX-2843. For the inhibitor condition, BMDMs were treated with 150 nM MRX-2843 for 2 h immediately before the efferocytosis assay. Matched negative-control siRNA, empty vector, and vehicle were used whenever the corresponding gene-targeting reagent or inhibitor was absent. Bmal1 siRNA was transfected using Lipofectamine RNAiMAX, whereas the Bmal1 and Mertk expression plasmids were transfected using Lipofectamine 2000. Knockdown or overexpression and Mertk restoration were verified before the efferocytosis assay. For the efferocytosis assays, BMDMs were isolated separately from three donor mice. For each condition, cells from each donor were maintained in a separate culture dish, and each donor-specific dish constituted one biological replicate (n = 3 per condition).

Jurkat cells in logarithmic growth were collected at 1 × 10⁶ cells/mL, washed once with sterile PBS, and gently resuspended in prewarmed phosphate-buffered saline (PBS) containing 5 μM CFDA-SE. After incubation for 15 min at 37 °C in the dark, the cells were pelleted, resuspended in fresh complete Roswell Park Memorial Institute 1640 medium (RPMI-1640), and incubated for a further 30 min at 37 °C to allow intracellular processing of the tracer. The labeled cells were then washed twice with sterile PBS and treated with 1 μM dexamethasone for 7 h to generate apoptotic target cells [47, 48]. Before co-culture, the cells were centrifuged, the dexamethasone-containing medium was removed, and the targets were washed three times with sterile PBS and resuspended in dexamethasone-free complete medium. BMDMs subjected to the indicated ox-LDL or siRNA interventions were labeled in situ with CellTracker Red CMTPX. Thus, dexamethasone was used only during preparation of Jurkat target cells and was absent from the macrophage-target co-culture.

CFDA-SE-labeled apoptotic Jurkat cells were added to CellTracker Red-labeled BMDMs at a target-to-phagocyte ratio of 5:1 and incubated for 2 h at 37 °C in the dark. The co-cultures were washed three times with sterile PBS to remove free apoptotic cells and fixed in 4% paraformaldehyde for 15 min. Quantification was performed by fluorescence microscopy rather than flow cytometry. For each condition and donor-specific culture dish, two completely nonoverlapping fields were selected at random and acquired under identical settings. The efferocytosis index was calculated as the number of red macrophages containing at least one internalized green apoptotic target cell divided by the total number of red macrophages × 100% [41]. Each donor-specific dish represented one biological replicate, and the two fields acquired from each dish were retained as separate field-level measurements, yielding six plotted points from three donor mice per condition.

### Promoter mutagenesis and dual-luciferase reporter assay

Multiple potential Bmal1-binding motifs in the upstream regulatory region of the mouse Mertk gene were predicted using JASPAR [49] and ranked by relative profile score. The two highest-scoring candidate sites were selected for reporter analysis: candidate site 1 at − 763 to − 754 and candidate site 2 at − 111 to − 101 relative to the transcription start site. WT1-Mertk contained …CAACGTGAC…, whereas MUT1-Mertk contained …TGGTACAGT…. WT2-Mertk contained …GGCCACGCGT…, whereas MUT2-Mertk contained …AATTGTATAC…. The matched mutations disrupted the core sequence of each predicted Bmal1-binding motif while preserving promoter-fragment length.

Sequence-verified pGL3-Basic reporters containing the wild-type or matched mutant Mertk promoter fragments and the pcDNA3.1(+)-Bmal1 expression plasmid were obtained from GeneCreate (Wuhan, China). In HEK293T cells, WT1, MUT1, WT2, and MUT2 reporters were tested in parallel with the Bmal1 expression plasmid or matched empty vector. A complementary BMDM reporter experiment used four conditions: pGL3-Basic + siNC, pGL3-Basic + siBmal1, WT1 + siNC, and WT1 + siBmal1. Reporter and expression or siRNA reagents were introduced using Lipofectamine 2000 or Lipofectamine RNAiMAX, as appropriate, and a Renilla reporter was included as the internal transfection control. Cells were lysed 48 h after reporter transfection, firefly and Renilla activities were measured using the Dual-Luciferase Reporter Assay Kit, and promoter activity was calculated as the firefly/Renilla ratio before normalization to the corresponding control. These experiments tested the effects of Bmal1 abundance and mutation of the two predicted candidate sites on Mertk promoter activity.

### Cleavage under targets and release using nuclease assay

Cleavage under targets and release using nuclease followed by quantitative PCR (CUT&RUN-qPCR) was performed to examine Bmal1 occupancy at the Mertk promoter in BMDMs. Assays were performed using the Hyperactive pG-MNase CUT&RUN Assay Kit for PCR/qPCR (Cat# HD103; Vazyme Biotech, Nanjing, China) according to the manufacturer's instructions. Approximately 5 × 10^5^ BMDMs were used per reaction. Cells were incubated overnight at 4 °C with either 1 μg rabbit IgG isotype-control antibody (Cat# HA722127; Hangzhou HuaAn Biotechnology, Hangzhou, China) or 1 μg anti-Bmal1 polyclonal antibody (Cat# 14,268–1-AP; Proteintech, Wuhan, China). Antibody-bound chromatin was subjected to pG-MNase-mediated cleavage and DNA release, and the released DNA fragments were purified using the DNA-purification buffers and spin columns supplied with the kit.

Purified CUT&RUN DNA was analyzed by real-time quantitative PCR using ChamQ SYBR qPCR Master Mix (Cat# Q711; Vazyme Biotech) on an ABI ViiA 7 Real-Time PCR System (Thermo Fisher Scientific). Primers targeting the tested Mertk promoter region were forward, 5′-ATTCGGCTTAGTTCTCCCCG-3′, and reverse, 5′-TGGCTACTGAAACGGAGTGC-3′. Relative enrichment of the Mertk promoter was calculated by normalizing the anti-Bmal1 CUT&RUN signal to the IgG control, with the IgG group set to 1.

### RNA extraction and quantitative real-time PCR

Total cellular RNA was extracted using TRIzol reagent. One microgram of total RNA was reverse-transcribed into cDNA using the HiScript II 1 st Strand cDNA Synthesis Kit. qPCR amplification was subsequently performed on a LightCycler 480 Real-Time PCR System (Roche Diagnostics, Basel, Switzerland) using the AceQ qPCR SYBR Green Master Mix. The relative expression levels of target genes were quantified using the standardized calculation method in the Roche LightCycler 480 software and normalized against β-actin as the internal reference gene. The specific sequences of primers used for the qPCR analysis are detailed in Supplementary Table 8.

### Transcriptome sequencing and data processing

Total RNA from aortic tissues and cultured cells that passed quality assessment was used for library construction. Paired-end libraries were sequenced on an Illumina NovaSeq 6000 by OE Biotech (Shanghai, China). Reads were filtered with fastp v0.20.1 (–length_required 50) [50], aligned to GRCm39 with HISAT2 v2.1.0 [51], and counted with HTSeq-count v0.11.2 [52]. Differential expression was analyzed with DESeq2 [53]; genes with an absolute fold change ≥ 2 and a Benjamini–Hochberg false-discovery-rate-adjusted P < 0.05 were considered differentially expressed. KEGG [54] and GSEA [55] were used for pathway-level interpretation.

### Population-level transcriptome correlation analysis

We obtained large-scale whole-blood transcriptome sequencing data of human samples from the Genotype-Tissue Expression database (https://gtexportal.org/) via the GEPIA web server (http://gepia.cancer-pku.cn/). After the TPM (Transcripts Per Million) expression matrix of the target genes was extracted, Spearman correlation analysis was performed to evaluate the association between the expression levels of the human homologs of Bmal1 and Mertk. Data visualization and related statistical tests were performed via the GEPIA web server.

### Genome-wide association study structural equation modeling (GWAS-SEM) analysis

The same harmonized vascular-trait summary statistics were processed with GenomicSEM [34, 56]. Multivariable LD score regression was used to estimate the genetic covariance matrix and its sampling covariance matrix; off-diagonal sampling covariances incorporated the cross-trait LDSC intercepts so that covariance caused by overlapping participants was not treated as independent information. A single-factor model was fitted by diagonally weighted least squares. This latent factor was termed circulatory plaque burden (CPB) and integrated the shared genetic covariance of carotid plaque, coronary artery disease, peripheral artery disease, and abdominal aortic phenotypes. Model fit was evaluated using the chi-square statistic, comparative fit index, and standardized root mean square residual, together with the standardized factor loadings; the complete values are reported in Supplementary Table 2. Common-factor GWAS then estimated variant associations with CPB, followed by SNP2GENE annotation in Functional Mapping and Annotation (FUMA) [58] using P < 5 × 10⁻⁸ as the genome-wide threshold.

### Overall genetic correlation analysis

All input GWAS summary datasets listed in Supplementary Table 1 were derived from participants of European ancestry. Summary statistics were harmonized to the same effect allele, and variants were retained when INFO > 0.9 and minor-allele frequency > 0.01; the extended major histocompatibility complex region on chromosome 6 (25–34 Mb) was excluded. Global genetic correlations were estimated by linkage disequilibrium score regression [56] and high-definition likelihood [57] using ancestry-matched linkage disequilibrium information from the 1000 Genomes Project Phase 3 European reference panel. Cross-trait LD score regression intercepts were used to retain the sampling covariance attributable to participant overlap between GWAS datasets. These analyses tested whether fasting duration and CPB shared genome-wide genetic architecture and were not interpreted as estimating the effect of a fasting intervention.

### Pleiotropic association analysis

Shared genetic signals between fasting duration and CPB were evaluated using pleiotropic analysis under a composite null hypothesis (PLACO) [59] and Fisher's combined probability method. Signals meeting P < 5 × 10⁻⁸ were mapped with iCPAGdb [60] using the 1000 Genomes reference panel [61] and subjected to pathway enrichment. This analysis was used to identify pleiotropic loci and shared pathways; it did not estimate the direction or magnitude of a fasting intervention effect.

### Gene-level association analysis and transcriptome-wide association study (TWAS)

Multi-marker Analysis of GenoMic Annotation (MAGMA) [62] tested gene-level associations across protein-coding genes and their ± 10 kb regions, with significance controlled by Bonferroni correction across the genes tested. FUSION transcriptome-wide association framework [63] integrated Genotype-Tissue Expression (GTEx) project version 8 [64] expression quantitative-trait-locus weights for transcriptome-wide association analysis. These analyses prioritized genes whose inherited association or genetically predicted expression converged with CPB-related signals; TWAS associations were not treated as functional confirmation or standalone causal evidence.

### Proteome-wide mendelian randomization (PW-MR)

Plasma protein quantitative trait loci (pQTLs) from the deCODE cohort [65] were selected within a 1 Mb window at P < 5 × 10⁻⁸. Proteome-wide Mendelian randomization used the inverse-variance-weighted estimator, with significance assessed using Bonferroni correction across the proteins tested. Estimates were interpreted as genetically supported directional associations conditional on the relevance, independence, and exclusion-restriction assumptions for the instruments.

### Fine-mapping analysis

Fine-mapping Of CaUsal gene Sets (FOCUS) [66] estimated gene-level posterior inclusion probabilities from the expression-correlation covariance matrix. At the variant level, Sum of Single Effects (SuSiE) [67] generated 95% credible sets with a maximum of 10 causal variants, and FINEMAP [68] calculated posterior probabilities for cross-validation using the same maximum. Fine-mapping outputs were used to narrow candidate genes and variants within associated regions and were not interpreted as functional validation.

### Single-cell transcriptome processing, macrophage-state trajectory, and virtual network perturbation analysis

Single-cell datasets GSE314596, GSE184073, and GSE253903 were processed independently in R version 4.6.0. The GSE253903 single-cell RNA-sequencing subset comprised 12 carotid endarterectomy specimens, including six from clinically symptomatic patients. In the source cohort, symptomatic status was defined by stroke or transient ischemic attack within 6 months before surgery, whereas asymptomatic patients had no history of ischemic events. For GSE184073, CD45-positive plaque cells from four patients with chronic coronary syndrome/stable angina pectoris whose plaques lacked a high-intensity signal on non-contrast T1-weighted magnetic resonance imaging and three patients with acute coronary syndrome were pooled within each clinical group before single-cell RNA sequencing. We therefore refer to these as chronic-coronary-syndrome/stable-angina-associated and acute-coronary-syndrome-associated plaque groups, rather than as histologically defined stable and unstable plaques. Because cells were nested within patients and donor identity was not retained after pooling in GSE184073, clinical-group comparisons were interpreted as descriptive cell-level evidence rather than donor-level inference. Genes detected in fewer than three cells were removed. Cells were retained when they contained 50–6,000 detected genes, 100–50,000 unique molecular identifier counts, and < 20% mitochondrial transcripts. Doublets were identified separately within each dataset using DoubletFinder version 2.0.4 [69], with pN = 0.25, pK selected by the maximum mean–variance-normalized bimodality coefficient for that dataset, an expected doublet rate of 7.5%, and adjustment for the estimated homotypic-doublet proportion. After quality control, counts were normalized with LogNormalize using a scale factor of 10,000 in Seurat version 4.4.0 [70]. The 2,000 most variable genes were selected by the variance-stabilizing transformation method, followed by scaling and principal-component analysis. Harmony version 1.2.0 (Korsunsky et al., 2019) was applied to the principal-component space to reduce sample-level batch effects. The first 30 Harmony dimensions were used to construct the shared-nearest-neighbor graph, perform Louvain clustering at resolution 0.6, and generate uniform manifold approximation and projection (UMAP) coordinates. Monocle 3 [71] and scTenifoldKnk [35] were used for the downstream macrophage-state and virtual-perturbation analyses described below.

Cluster-enriched genes were identified with a Wilcoxon rank-sum test using a minimum detection fraction of 0.25, absolute log2 fold change > 0.25, and Benjamini-Hochberg-adjusted P < 0.05. Cell identities were assigned manually from combinations of canonical lineage markers, with the marker evidence for each cluster checked individually against the curated human marker records in CellMarker 2.0 [72]. The macrophage subset was transferred to monocle3 version 1.3.7, a principal graph was learned in the UMAP space, and cells were ordered along the graph. The efferocytosis module score was calculated from the normalized expression of representative apoptotic-cell recognition and clearance genes, including Mertk, AXL, GAS6, MFGE8, TIMD4, STAB1, and LRP1. The inflammation module score was calculated in the same manner from representative inflammatory genes, including IL1B, TNF, CCL2, CXCL8, NFKB1, and PTGS2. The 50 macrophages with the highest inflammation module scores were specified as root cells, defining the high-inflammation end of each inferred trajectory. For Fig. 5A and C, normalized expression of the Bmal1 and Mertk homologs and the efferocytosis module score were fitted along the rescaled pseudotime axis. Cell-level Pearson correlations between normalized expression of the Bmal1 and Mertk homologs were calculated separately for macrophages from normal coronary arteries, coronary artery plaques, asymptomatic carotid plaques, and symptomatic carotid plaques. The cell-level Mertk homolog expression distributions in stable and unstable coronary plaques were compared using a two-sided Wilcoxon rank-sum test.

For virtual perturbation, scTenifoldKnk constructed five macrophage gene-regulatory subnetworks with tensor rank 2 and simulated knockout of the human homolog of Bmal1 by setting its outgoing regulatory weights to zero. Network-distance values were Box-Cox transformed and standardized within each dataset. The resulting Z score is a within-dataset measure of how strongly a gene's network relationships deviate from the unperturbed state: a larger value indicates a more pronounced relative network disturbance after virtual knockout of the Bmal1 homolog. It does not indicate whether the gene is transcriptionally upregulated or downregulated, and values were not compared quantitatively across different datasets. Cell-level maps were used for visualization, whereas cell-type summaries and phenotype comparisons were aggregated at the patient or sample level for statistical inference. Virtual perturbation was performed independently of the Monocle 3 pseudotime analysis, so candidate ranking did not depend on trajectory rooting or pseudotime ordering. The model assumes that the expression-derived network captures biologically relevant regulatory structure and that setting outgoing Bmal1-homolog weights to zero approximates loss of regulatory activity; accordingly, the output was used for hypothesis generation rather than interpreted as an experimental knockout or causal test. Robustness was assessed by repeating the analysis in three independently processed arterial macrophage datasets and prioritizing only shared candidates. Formal sensitivity analyses using alternative clustering, batch-correction, or network-parameter settings were not performed.

### Randomization, blinding, experimental units, and statistical analysis

Statistical analyses were performed in R version 4.6.0. After group allocation, animal, serum, and tissue samples were assigned coded identifiers, and the order of biochemical measurement, section selection, staining, image acquisition, and initial image quantification was interspersed across groups. Group labels remained concealed during these prespecified measurements and initial quantification. After all prespecified measurements had been completed, the code key was opened by the sole first author, who assembled the final dataset and performed the final statistical analysis and integration. The mouse was the independent unit for animal experiments, and a donor-specific BMDM culture or independently repeated cell experiment was the unit for in vitro analyses. For tissue analyses, measurements from multiple sections or fields were averaged within the corresponding mouse. For the BMDM efferocytosis assays, the two field-level measurements from each donor-specific dish were retained and displayed separately as nested measurements, yielding six points from three donor mice per condition. Data are presented as mean ± standard error of the mean (SEM) unless otherwise stated.

Before parametric testing, normality of animal and cell outcome data was assessed using the Shapiro–Wilk test and homogeneity of variance using Levene's test. Two independent groups satisfying parametric assumptions were compared by an unpaired two-tailed Student's t test; three or more groups were compared by one-way ANOVA followed by Tukey's multiple-comparison test. Outcomes not satisfying parametric assumptions were analyzed using the corresponding prespecified nonparametric test. Longitudinal body-weight and food-intake data were analyzed with a two-way repeated-measures framework that included group, time, and their interaction. Exact biological sample sizes are reported for the corresponding outcomes, and in vitro results were derived from at least three independent biological replicates. The cell-level expression comparisons in Fig. 3H and Supplementary Fig. 6B used two-sided Wilcoxon rank-sum tests. Exact P values are displayed in the relevant panels when available; otherwise, the figure legends define the prespecified significance thresholds. Statistical tests and multiple-comparison procedures are specified in the corresponding legends. Package-specific models were used for other transcriptomic, single-cell, and population-genetic analyses [73, 74].

CUT&RUN-qPCR group comparisons were performed on ΔCt values using a paired two-sided Student's t test. Normalization of the IgG signal to 1 was used only for graphical presentation of relative promoter enrichment.

For bulk RNA sequencing, differential expression was controlled at Benjamini–Hochberg FDR-adjusted P < 0.05. GWAS and PLACO analyses used P < 5 × 10⁻⁸; MAGMA and proteome-wide Mendelian randomization used analysis-specific Bonferroni correction; and Bayesian fine-mapping was summarized by posterior inclusion probabilities, posterior probabilities, and 95% credible sets. For other prespecified experimental analyses, two-sided P < 0.05 was considered statistically significant.

## Results

### GWAS-SEM constructs a systemic plaque burden index and links it to nutrient-responsive processes

**Fig. 1:**
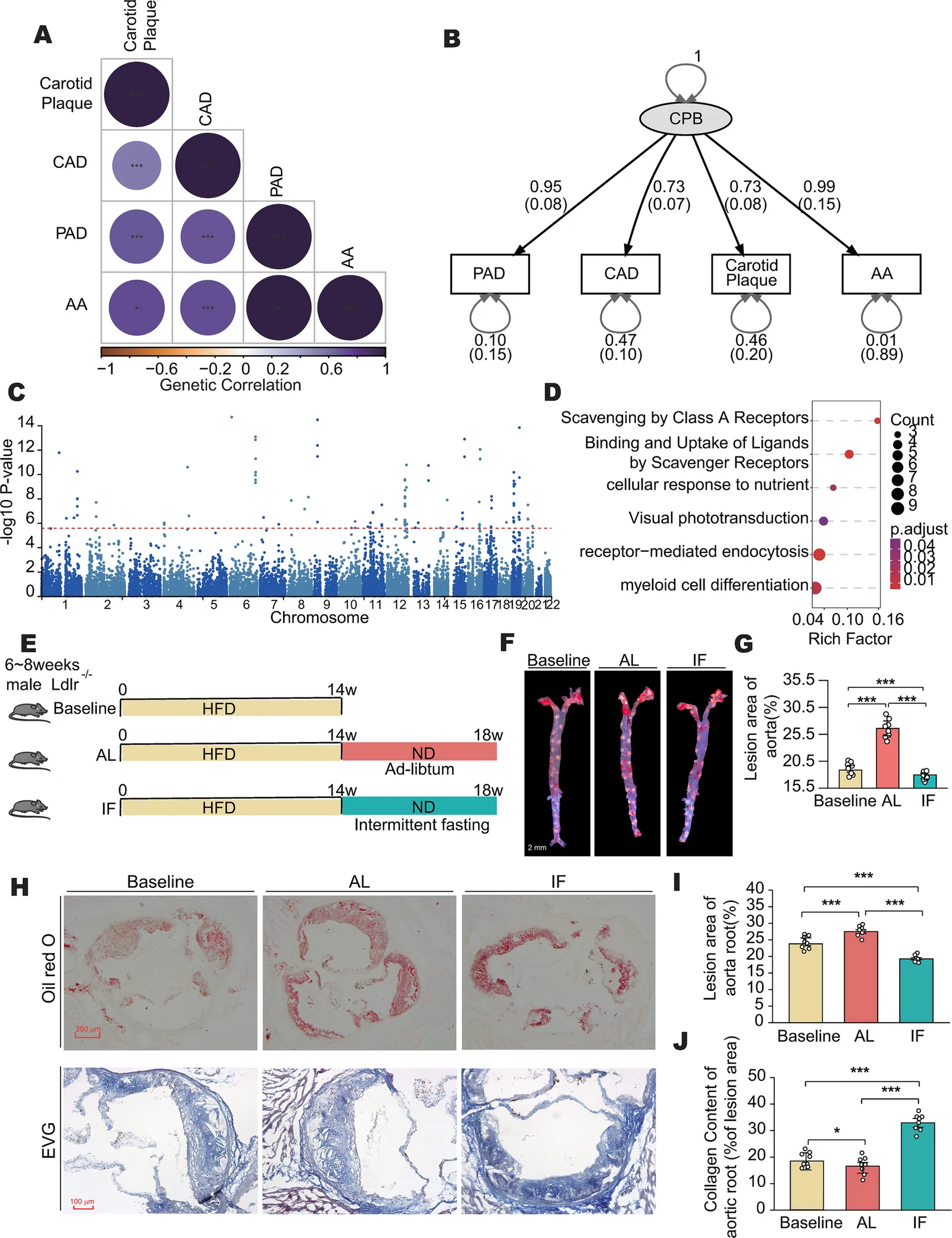
Genetic construction of circulatory plaque burden and plaque remodeling with intermittent fasting. **A** Pairwise genome-wide genetic correlations among carotid plaque, coronary artery disease (CAD), peripheral artery disease (PAD), and abdominal aortic (AA) phenotypes; color and circle size represent the magnitude of the genetic correlation. **B** Single-factor genome-wide association study structural equation model (GWAS-SEM) used to construct circulatory plaque burden (CPB), a latent variable representing shared inherited liability across the four vascular phenotypes. Values on the paths are standardized factor loadings, with standard errors in parentheses. Model fit: comparative fit index = 0.999, standardized root mean square residual = 0.030, χ.^2^ = 0.442, P = 0.802. **C** Manhattan plot of the common-factor GWAS for CPB. The red dashed line marks the genome-wide significance threshold (P < 5 × 10⁻⁸). **D** Functional enrichment of genes prioritized from CPB-associated loci; bubble size indicates the number of genes and color indicates the adjusted *P* value. **E** Experimental design. Eight-week-old male Ldlr⁻/⁻ mice received a high-fat, high-cholesterol diet (HFD) for 14 weeks. A randomly selected group was euthanized at week 14 as the intervention-start Baseline; the remaining mice were switched to a normal diet (ND) and assigned to ad libitum feeding (AL) or intermittent fasting (IF), defined as alternating 24 h of complete fasting with water available and 24 h of ad libitum feeding, for 4 weeks. **F**, **G** Representative en face Oil Red O staining of the whole aorta (**F**) and lesion area as a percentage of total intimal surface area (**G**). Scale bar, 2 mm. **H**–**J** Representative Oil Red O and Elastica van Gieson (EVG) staining of level-matched aortic-root sections (H), with quantification of plaque area (**I**) and collagen content (**J**). Scale bars, 250 μm (Oil Red O) and 100 μm (EVG). For F–J, n = 8 mice per group, and each point represents one mouse. Data are mean ± SEM. Group comparisons in G, I, and J used one-way ANOVA followed by Tukey's multiple-comparison test. All reported animal-comparison P values are two-sided. *P < 0.05; ***P < 0.001

To examine the shared genetic basis of atherosclerotic lesions across vascular territories, we first assessed genetic correlations among carotid plaque, coronary artery disease (CAD), peripheral artery disease (PAD), and abdominal aortic (AA) phenotypes. All six pairwise correlations among the four traits were positive and statistically significant (Fig. 1A). We therefore integrated these traits into a latent variable termed circulatory plaque burden (CPB) using genome-wide association study structural equation modeling (GWAS-SEM) [34, 56]. The model showed a good fit (comparative fit index = 0.999, standardized root mean square residual = 0.030, *χ*^*2*^ = 0.442, *P* = 0.802), with standardized factor loadings ranging from 0.73 to 0.99 (Fig. 1B). A subsequent genome-wide association analysis of CPB identified multiple association loci exceeding the genome-wide significance threshold (*P* < 5 × 10⁻⁸; Fig. 1C). The input GWAS datasets and sample sizes are detailed in Supplementary Table 1, and the complete GenomicSEM parameter estimates are provided in Supplementary Table 2.

To map the SNP-level associations to genes and biological processes, we analyzed the CPB GWAS summary statistics using FUMA [58] and MAGMA [62]. MAGMA yielded 54 reported gene-level associations meeting the table-specific reporting threshold; these genes were subjected to functional enrichment analysis (Supplementary Table 4). The enriched pathways included scavenger-receptor-mediated ligand uptake, cellular responses to nutrient levels, receptor-mediated endocytosis, and myeloid cell differentiation (Fig. 1D). Together, these analyses defined a genetically informed index of systemic atherosclerotic involvement and highlighted nutrient-responsive processes among the biological pathways associated with plaque burden.

### Intermittent fasting reduces established plaque burden and improves features of plaque stability

Motivated by the genetic association with nutrient-responsive processes, we next examined the effect of intermittent fasting on established atherosclerotic lesions. Eight-week-old male Ldlr⁻/⁻ mice were fed a high-fat, high-cholesterol diet for 14 weeks. At the end of this induction period, one-third of the mice were randomly selected and euthanized as the week-14 Baseline group. The remaining mice were switched to a normal diet and randomly assigned to either an ad libitum-fed group or an alternate-day 24-h intermittent-fasting group for four weeks (Fig. 1E).

To assess group comparability at week 14, serum lipid levels and body weight were measured after the 14-week induction period and before the four-week feeding intervention. No significant differences in total cholesterol (TC), triglycerides (TG), low-density lipoprotein cholesterol (LDL-C), high-density lipoprotein cholesterol (HDL-C), non-esterified fatty acids (NEFA), or body weight were detected among mice allocated to the Baseline, ad libitum-fed, and intermittent-fasting groups (Supplementary Fig. 1A–F; all P > 0.05), supporting balance in the assessed systemic variables at allocation.

Whole-aorta Oil Red O staining showed that lesion area was greater in the ad libitum-fed group than at Baseline. In contrast, the lesion area in the intermittent-fasting group was lower than that in the ad libitum-fed group and was also lower than that in the week-14 Baseline group (Fig. 1F, G). Oil Red O staining of aortic-root sections showed a consistent pattern, with a smaller plaque area in the intermittent-fasting group than in both the ad libitum-fed and Baseline groups (Fig. 1H, I). At the group level, plaque burden after intermittent fasting was lower than the week-14 Baseline reference, supporting regression of established atherosclerotic lesions.

Elastica van Gieson staining further showed that plaque collagen content was lower in the ad libitum-fed group than at Baseline, whereas intermittent fasting increased collagen content relative to both the ad libitum-fed and Baseline groups (Fig. 1H, J). Thus, the reduction in plaque burden induced by intermittent fasting was accompanied by increased collagen deposition, a structural feature associated with greater plaque stability.

**Fig. 2:**
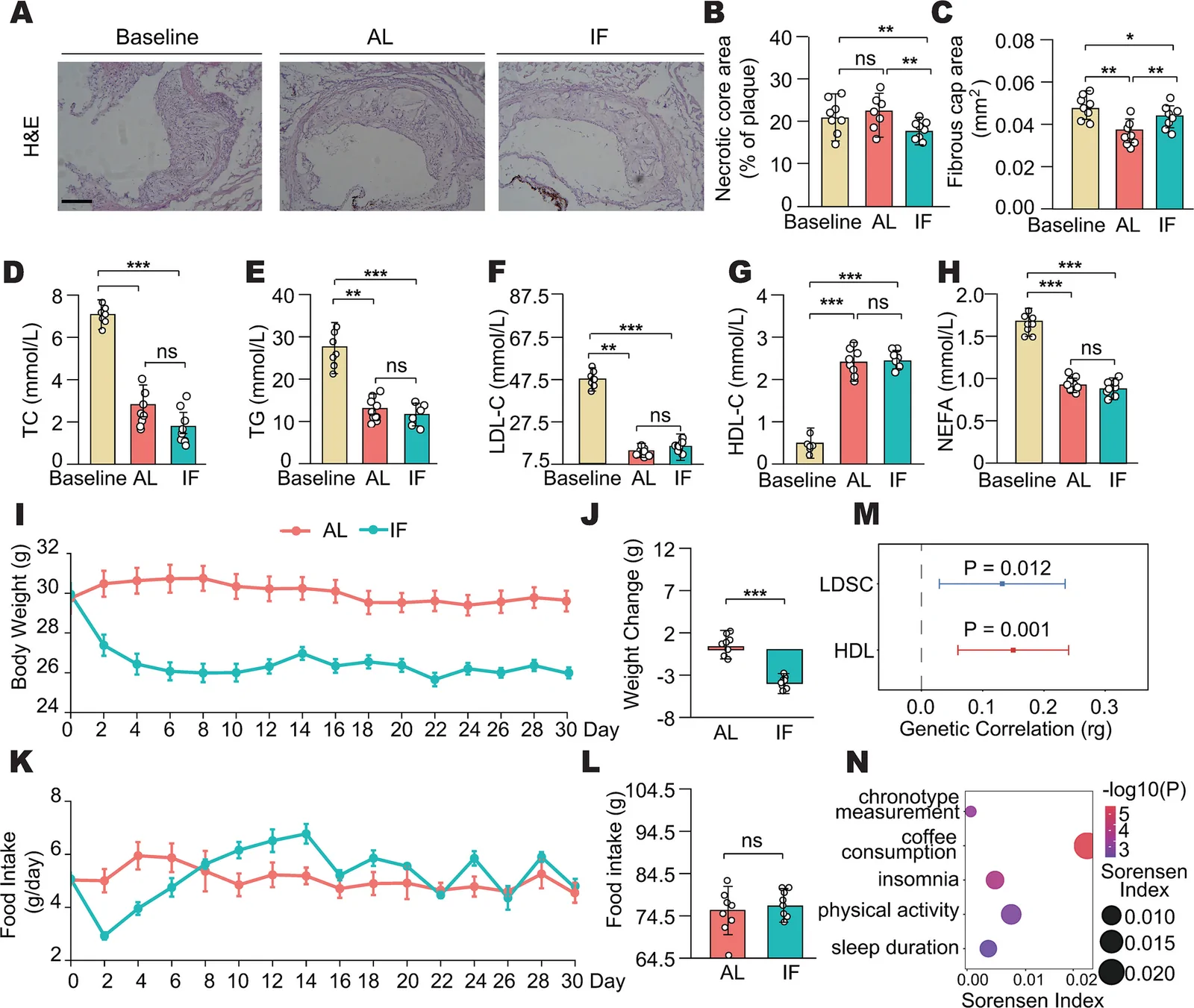
Plaque composition, systemic metabolic measures, and fasting-related genetic architecture. **A**–**C** Representative hematoxylin and eosin staining of level-matched aortic-root plaques (**A**) and quantification of necrotic-core area as a percentage of plaque area (**B**) and fibrous-cap area (**C**). Dashed lines delineate the necrotic core. Scale bar, 50 μm. NC, necrotic core; FC, fibrous cap. **D**–**H** Serum total cholesterol (TC), triglycerides (TG), low-density lipoprotein cholesterol (LDL-C), high-density lipoprotein cholesterol (HDL-C), and non-esterified fatty acids (NEFA) at the intervention-start Baseline or after the 4-week AL or IF intervention. **I**, **J** Body-weight trajectory and net weight change during the intervention. (**K**, **L**) Food intake over time and cumulative food intake. For A–H, n = 8 mice per group; for I–L, n = 8 mice per AL or IF group. **M** Genetic correlation of fasting duration with CPB estimated by linkage disequilibrium score regression (LDSC) and high-definition likelihood (HDL); exact P values are shown. Error bars denote 95% confidence intervals. **N** Enrichment of phenotypes linked to pleiotropic loci shared by fasting duration and CPB; bubble size indicates the Sørensen index and color indicates − log10(P). Data in B–L are mean ± SEM. B–H were analyzed by one-way ANOVA followed by Tukey's multiple-comparison test; I and K were analyzed using a two-way repeated-measures framework including group, time, and their interaction; J and L were analyzed by unpaired two-sided Student's t test. All reported animal-comparison P values are two-sided. *P < 0.05; **P < 0.01; ***P < 0.001; ns, not significant

Hematoxylin and eosin staining of aortic-root lesions was subsequently used to assess additional structural features of plaque composition (Fig. 2A). The necrotic-core fraction was lower in the intermittent-fasting group than in both the concurrent ad libitum-fed group and the week-14 Baseline group (both *P* < 0.01), whereas no significant difference was detected between the ad libitum-fed and Baseline groups (Fig. 2B). Fibrous-cap area was lower in the ad libitum-fed group than at Baseline (*P* < 0.01). Intermittent fasting increased the fibrous-cap area relative to ad libitum feeding (*P* < 0.01), although it remained lower than the Baseline value (*P* < 0.05; Fig. 2C). Together with the increased collagen content shown in Fig. 1, these findings demonstrate that intermittent fasting was associated with a smaller necrotic core and a greater fibrous component than ad libitum feeding, two structural features consistent with a more favorable plaque phenotype.

Plaque MMP9 and CCL2 signals provided complementary molecular readouts of this remodeling phenotype. Both signals were higher in the ad libitum-fed group than at the week-14 Baseline and were lower in the intermittent-fasting group than in both the ad libitum-fed and Baseline groups (Supplementary Fig. 2A–D). These changes accompanied the smaller necrotic core, greater fibrous-cap area, and higher collagen content observed with intermittent fasting, consistent with reduced matrix-degrading and monocyte-recruiting signals within plaque regions.

### Plaque remodeling with intermittent fasting occurs without detectable additional endpoint lipid lowering

At the four-week endpoint, TC, TG, LDL-C, and NEFA levels were lower, whereas HDL-C levels were higher, in both intervention groups than in the week-14 Baseline group (Fig. 2D–H). No significant difference between the concurrent ad libitum-fed and intermittent-fasting groups was detected for any of the five lipid variables. Thus, the differences in plaque burden and composition between the intermittent-fasting and ad libitum-fed groups occurred without a detectable additional reduction in endpoint circulating lipid levels in the intermittent-fasting group. Because the study did not include an age-matched chow-fed Ldlr⁻/⁻ group without prior high-fat, high-cholesterol diet exposure or a wild-type reference group, these measurements demonstrate lipid lowering relative to the week-14 Baseline but do not establish normalization to a nonatherogenic reference range.

Body weight decreased rapidly after initiation of intermittent fasting and remained lower throughout the intervention, resulting in a greater net weight loss than in the ad libitum-fed group (*P* < 0.001; Fig. 2I, J). In contrast, cumulative food intake over the four-week intervention did not differ significantly between the ad libitum-fed and intermittent-fasting groups (*P* > 0.05; Fig. 2K, L). Thus, intermittent fasting altered body weight despite comparable measured cumulative food intake.

Using published genome-wide association summary statistics for fasting duration, we next examined its shared genetic architecture with circulatory plaque burden (CPB). Significant genetic correlations were detected using both linkage disequilibrium score regression [56] (LDSC; *P* = 0.012) and high-definition likelihood [57] (HDL; *P* = 0.001; Fig. 2M). Analysis of pleiotropic loci shared by fasting duration and CPB further identified enrichment for chronotype measurement, coffee consumption, insomnia, physical activity, and sleep duration (Fig. 2N). Together with the CPB enrichment for myeloid differentiation, scavenger-receptor biology, and receptor-mediated endocytosis in Fig. 1D, these shared circadian and behavioral signals pointed to an intersection between clock regulation and macrophage function and prompted direct analysis of the aortic lesions.

### Transcriptomic analyses identify Bmal1 as a circadian regulator responsive to intermittent fasting in lesions

**Fig. 3:**
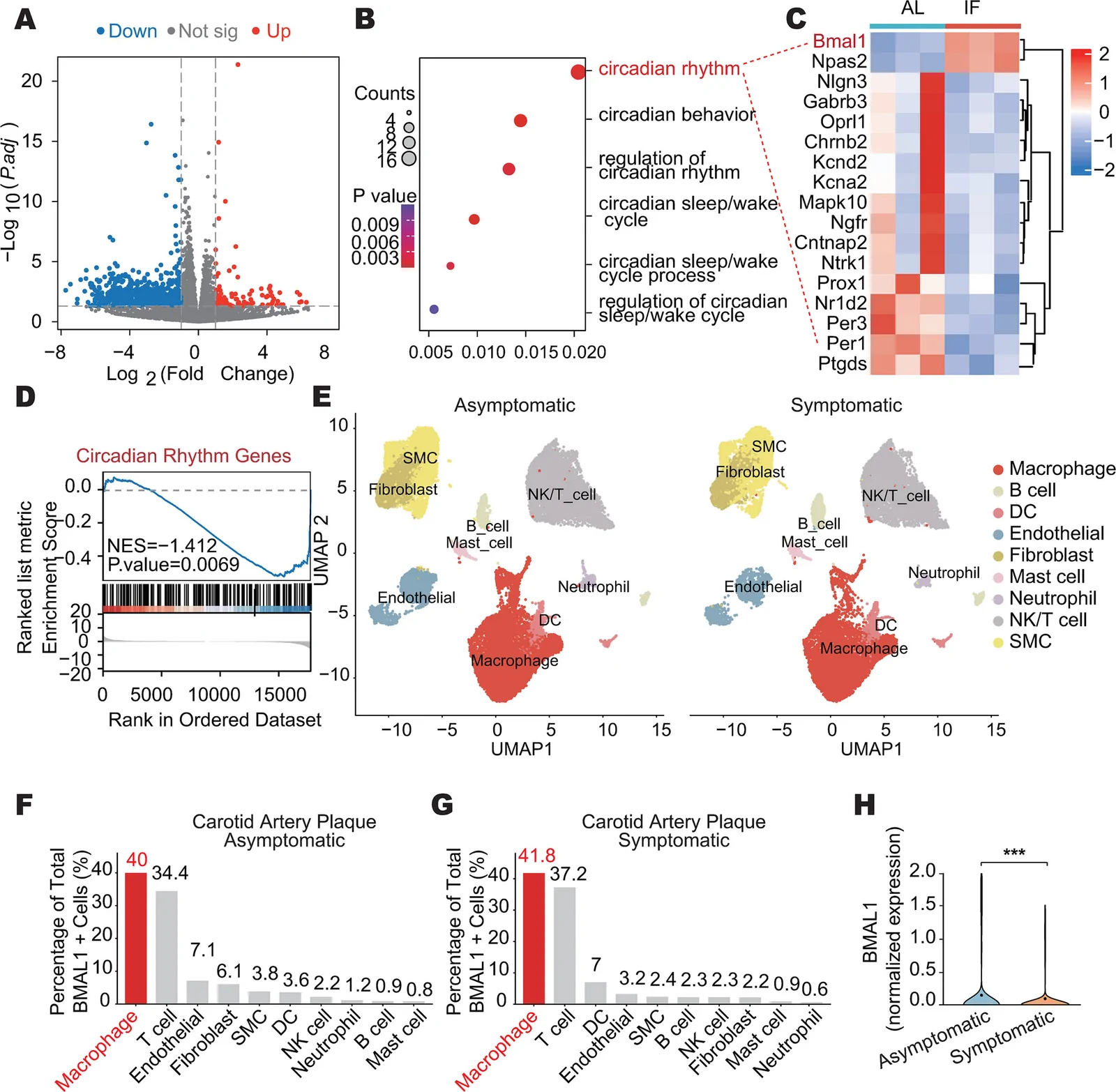
Aortic transcriptomics identifies an intermittent-fasting-responsive circadian program and human plaque single-cell data localize expression of the Bmal1 homolog. **A** Volcano plot of differentially expressed genes in plaque-bearing aortas from AL and IF mice (n = 3 mice per group). Colored points denote genes with an absolute fold change ≥ 2 and Benjamini–Hochberg false-discovery-rate-adjusted *P* < 0.05. **B** Enrichment of circadian-rhythm-related processes among the differentially expressed genes; bubble size indicates gene count and color indicates P value. **C** Row-scaled expression heatmap of selected circadian-rhythm-related genes in AL and IF aortas (n = 3 mice per group), highlighting Bmal1 and Npas2. **D** Gene set enrichment analysis of the circadian-rhythm gene set (normalized enrichment score = − 1.412, P = 0.0069). **E** Uniform manifold approximation and projection (UMAP) of major immune and vascular populations in asymptomatic and symptomatic human carotid plaques from GSE253903 (12 carotid endarterectomy specimens; 6 clinically symptomatic). In the source cohort, symptomatic status was defined by stroke or transient ischemic attack within 6 months before surgery, whereas asymptomatic patients had no history of ischemic events. **F**, **G** Distribution of cells with detectable expression of the human Bmal1 homolog among annotated cell populations in asymptomatic (**F**) and symptomatic (**G**) plaques; macrophages comprised 40.0% and 41.8% of positive cells, respectively. **H** Cell-level expression distribution of the human Bmal1 homolog in asymptomatic and symptomatic plaques. The plotted comparison used a two-sided Wilcoxon rank-sum test; ***P < 0.001. Because cells are nested within donors and symptom status is not a direct histological classification of plaque stability, H is presented as descriptive cell-level evidence rather than donor-level statistical inference. DC, dendritic cell; SMC, smooth muscle cell

To investigate the molecular changes associated with intermittent-fasting-induced plaque remodeling, we performed whole-transcriptome RNA sequencing of aortic tissues from the ad libitum-fed and intermittent-fasting groups. Differential expression analysis identified broad transcriptional differences between the two groups (Fig. 3A). Functional enrichment analysis of the differentially expressed genes highlighted circadian-rhythm-related pathways, including circadian rhythm, circadian behavior, regulation of circadian rhythm, and regulation of the circadian sleep/wake cycle (Fig. 3B). Gene set enrichment analysis [55] further identified significant differential enrichment of the circadian rhythm gene set between the ad libitum-fed and intermittent-fasting groups (normalized enrichment score = − 1.412, *P* = 0.0069; Fig. 3D). Together, these analyses identified circadian regulation as a prominent component of the aortic transcriptional response to intermittent fasting.

Examination of the circadian-rhythm-related genes revealed a selective rather than uniform transcriptional response to intermittent fasting. The core clock factors Bmal1 and *Npas2* were both significantly upregulated in the intermittent-fasting group relative to the ad libitum-fed group (Fig. 3C). Bmal1 was prioritized over Npas2 because it is a central core-clock component with established macrophage-intrinsic immune and metabolic functions, providing a direct mechanistic bridge from the lesion circadian signal to the macrophage/efferocytosis hypothesis. This prioritization did not imply that Npas2 was biologically irrelevant; its role was not tested in the present study.

We then examined transcripts corresponding to the human homolog of Bmal1 in single-cell RNA-sequencing data from GSE253903. The analyzed subset comprised 12 carotid endarterectomy specimens, six from clinically symptomatic patients. Major immune and vascular populations were identified in both clinical groups (Fig. 3E). Among cells with detectable expression, macrophages constituted the largest population in asymptomatic and symptomatic plaques, accounting for 40.0% and 41.8%, respectively, followed by T-lineage cells (Fig. 3F, G). The plotted cell-level expression distribution was higher in asymptomatic than symptomatic plaques (Fig. 3H). Because clinical symptom status reflected recent stroke or transient ischemic attack rather than a direct histological classification of plaque stability, we interpret this finding as supporting a potential association between higher plaque Bmal1 expression and an asymptomatic clinical phenotype.

Plaque immunofluorescence provided tissue-level support for this macrophage-linked Bmal1 response. Relative Bmal1 fluorescence within CD68-positive plaque regions was lower in the ad libitum-fed group than at the week-14 Baseline and was higher in the intermittent-fasting group than in both groups (Supplementary Fig. 3 A, B).

Co-immunofluorescence staining was next used to assess the anti-inflammatory cytokine IL10 in the context of the plaque macrophage marker F4/80. IL10 signal was lower in the ad libitum-fed group than at the week-14 Baseline and was higher in the intermittent-fasting group than in the ad libitum-fed group, although it remained below the Baseline value. Conversely, F4/80 signal was higher in the ad libitum-fed group than at Baseline and was lower in the intermittent-fasting group than in both groups (Supplementary Fig. 4A–C). The recovery of IL10 signal together with reduced F4/80 signal supports an intermittent-fasting-associated shift toward a less inflammatory macrophage-associated plaque phenotype.

### Single-cell virtual network perturbation identifies mertk as a candidate downstream effector of Bmal1

**Fig. 4:**
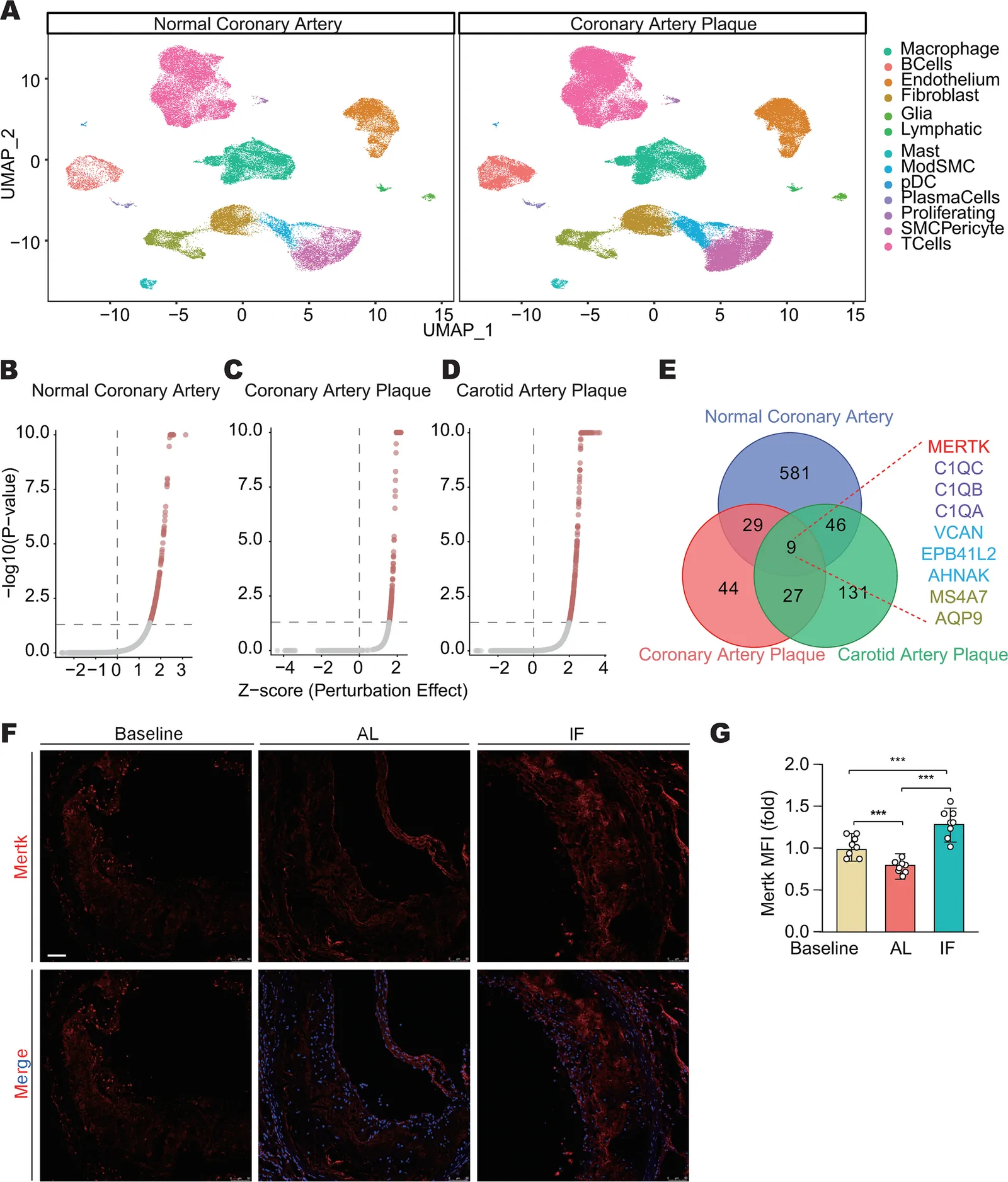
Single-cell virtual network perturbation prioritizes the Mertk homolog as a candidate Bmal1 effector, and intermittent fasting is associated with increased plaque-region Mertk signal. **A** UMAP representation of major immune and vascular populations in normal human coronary arteries and coronary artery plaques from GSE314596. **B**–**D** scTenifoldKnk virtual knockout of the human Bmal1 homolog in macrophages from normal coronary arteries (**B**), coronary artery plaques (C), and carotid artery plaques (D; GSE253903). Network-distance values were standardized within each dataset. A larger positive Z score indicates a greater relative deviation of a gene's network relationships from the unperturbed network; it does not indicate the direction of expression change, and Z scores should not be compared quantitatively across datasets. The horizontal dashed line denotes nominal P = 0.05 and the vertical dashed line denotes Z = 0. **E** Intersection of genes significantly perturbed in the three virtual-knockout analyses, identifying nine shared candidates including the Mertk homolog. **F**, **G** Representative single-marker immunofluorescence images of Mertk in aortic-root plaques (**F**) and relative plaque-region mean fluorescence intensity (MFI; integrated plaque-region fluorescence divided by plaque-region area; G). Mertk is shown in red and nuclei in blue. Scale bar, 50 μm (applies to all images in **F**). Signal was quantified only within the prespecified plaque region; the outer arterial wall and elastic lamina were excluded, and measurements from three level-matched sections were averaged to yield one value per mouse (n = 8 mice per group). The single-marker measurement was not interpreted as macrophage-specific abundance. Data are mean ± SEM. Group comparisons in G used one-way ANOVA followed by Tukey's multiple-comparison test. All reported animal-comparison P values are two-sided. ***P < 0.001. Panel E is a set-intersection summary of shared candidate genes and is not a cell-by-cell comparison

Having identified Bmal1 as an intermittent-fasting-responsive circadian regulator in plaque-bearing aortas, we next sought to define its potential downstream network in human atherosclerotic lesions. We analyzed single-cell RNA-sequencing datasets from normal coronary arteries, coronary artery plaques, and carotid artery plaques. Uniform manifold approximation and projection delineated the major immune and vascular cell populations in normal coronary arteries and coronary plaques (Fig. 4A). We then performed an in silico knockout of the human homolog of Bmal1 in macrophages from each of the three arterial microenvironments using scTenifoldKnk [35]. This virtual knockout predicted extensive network perturbations in normal coronary arteries, coronary plaques, and carotid plaques (Fig. 4B–D), indicating that the inferred macrophage network is context dependent while retaining shared candidate downstream nodes across arterial beds and disease states.

Intersection of the genes significantly perturbed by this virtual knockout identified nine shared candidates across the three datasets, including the Mertk homolog (Fig. 4E). This gene encodes a TAM-family receptor tyrosine kinase that mediates apoptotic-cell recognition and efferocytosis in macrophages. Given the central role of defective efferocytosis in necrotic-core expansion and plaque destabilization, this gene was prioritized as a candidate downstream node for subsequent analyses. Thus, the cross-dataset virtual-perturbation analysis connected Bmal1-related network perturbation to a macrophage pathway directly relevant to the clearance of apoptotic cells.

Single-marker immunofluorescence showed lower plaque-region Mertk fluorescence in the ad libitum-fed group than in the week-14 Baseline group, whereas intermittent fasting increased the signal relative to both the ad libitum-fed and Baseline groups (Fig. 4F, G). Together with the cross-dataset virtual perturbation, this in vivo response prioritized Mertk as a candidate Bmal1-associated effector for subsequent macrophage-state and functional analyses.

### Macrophage-state analyses and plaque imaging support bmal1-mertk-associated efferocytosis

**Fig. 5:**
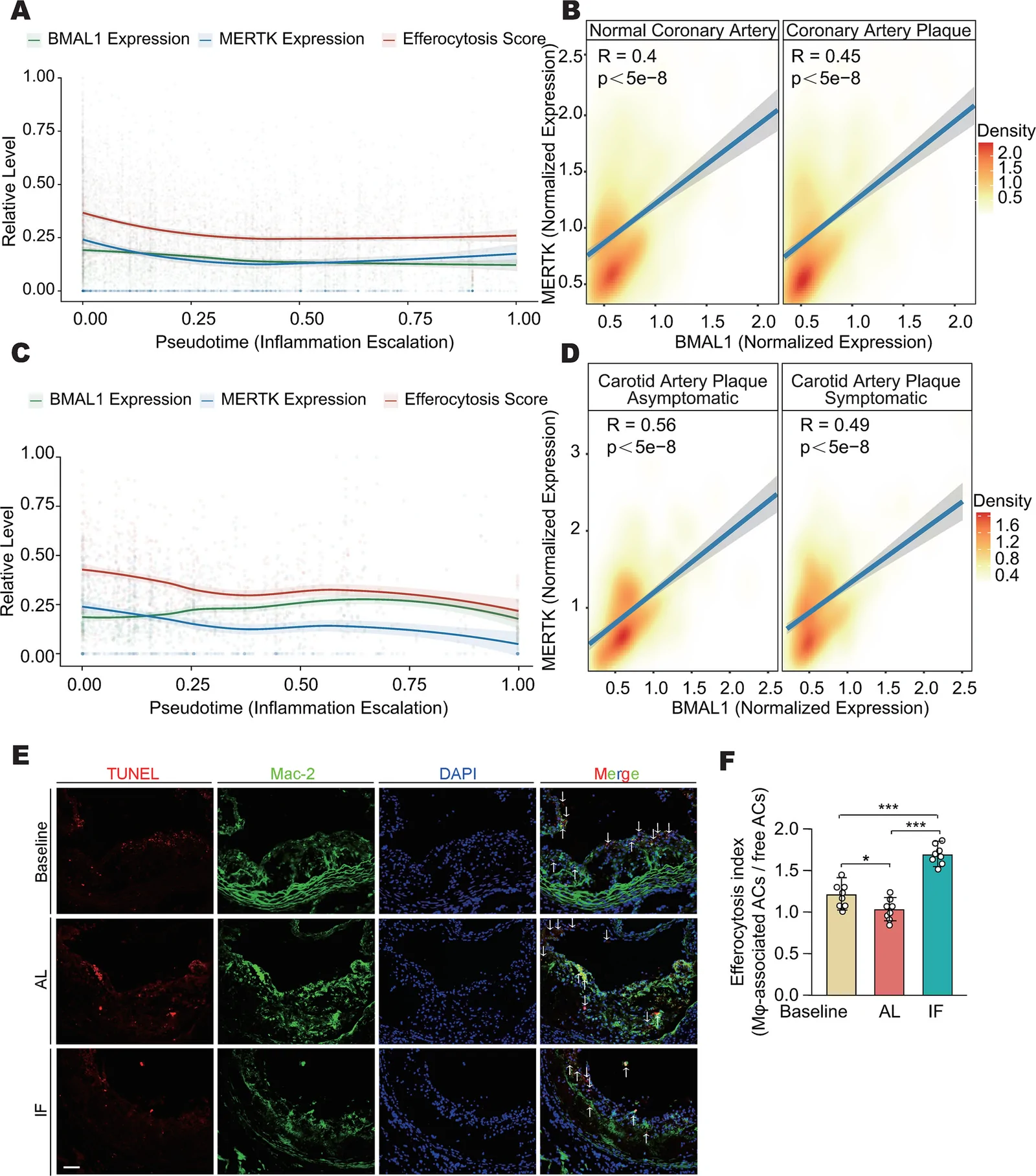
Human arterial macrophage-state analyses and aortic-root imaging support Bmal1-Mertk-associated efferocytosis. **A**, **C** Pseudotime trajectories of macrophages from human coronary artery microenvironments (A; GSE314596) and asymptomatic and symptomatic carotid plaques (C; GSE253903). Fitted curves show state-dependent variation in normalized expression of the Bmal1 homolog (green), normalized expression of the Mertk homolog (blue), and the efferocytosis module score (red); shaded bands denote confidence intervals. **B**, **D** Cell-level density plots and fitted linear relationships between normalized expression of the Bmal1 and Mertk homologs in macrophages from normal coronary arteries and coronary artery plaques (**B**), and from asymptomatic and symptomatic carotid plaques **D**. Pearson correlation coefficients (R) and P values are displayed in each panel. **E** Representative TUNEL and Mac-2 immunofluorescence images of aortic-root plaques from the week-14 Baseline, ad libitum-fed (AL), and intermittent-fasting (IF) groups. Apoptotic cells (ACs) are shown by TUNEL in red, macrophages by Mac-2 in green, and nuclei by DAPI in blue. In the merged images, upward-pointing arrows identify macrophage-associated ACs, including red ACs overlapping with Mac-2 signal (yellow) or being contacted and encircled by green Mac-2 signal; downward-pointing arrows identify free red ACs lacking adjacent or surrounding Mac-2 signal. Scale bar, 50 μm. **F** Histologic efferocytosis index, calculated as Mac-2-positive macrophage-associated ACs divided by free ACs. A higher ratio indicates greater macrophage association relative to free apoptotic-cell accumulation. n = 8 mice per group, and each point represents one mouse. Data are mean ± SEM and were analyzed by one-way ANOVA followed by Tukey's multiple-comparison test. The displayed correlation and animal-comparison *P* values are two-sided. *P < 0.05; ***P < 0.001. Mφ, macrophage

To examine whether the Bmal1-Mertk relationship extended across human arterial macrophage states, we analyzed macrophage trajectories from GSE314596 and GSE253903. In coronary artery and carotid plaque macrophages, expression of the Bmal1 and Mertk homologs and the efferocytosis module score showed coordinated state-dependent variation along the macrophage pseudotime trajectory (Fig. 5A, C). Cell-level expression of the two homologs was positively correlated in normal coronary arteries (R = 0.40), coronary artery plaques (R = 0.45), asymptomatic carotid plaques (R = 0.56), and symptomatic carotid plaques (R = 0.49; P < 5 × 10⁻⁸ for all comparisons; Fig. 5B, D). In GTEx whole-blood transcriptomes, the two homologs were also positively correlated (Spearman ρ = 0.66, P = 4.9 × 10⁻^44^; Supplementary Fig. 5). Across plaque macrophage states and whole blood, these concordant associations were consistent with Mertk as a candidate downstream component of the Bmal1-related program, which was then tested experimentally.

We next quantified the spatial relationship between macrophages and apoptotic cells in aortic-root plaques. TUNEL-positive apoptotic cells overlapping with or contacted and encircled by Mac-2-positive signal were classified as macrophage-associated, whereas TUNEL-positive cells lacking adjacent Mac-2 signal were classified as free apoptotic cells (Fig. 5E). The efferocytosis index, defined as the ratio of Mac-2-positive macrophage-associated apoptotic cells to free apoptotic cells, was lower in the ad libitum-fed group than in the week-14 Baseline group (P < 0.05) and higher in the intermittent-fasting group than in both the Baseline and ad libitum-fed groups (P < 0.001 for both comparisons; n = 8 mice per group; Fig. 5F). Thus, intermittent fasting was associated with fewer free apoptotic cells relative to macrophage-associated apoptotic cells within plaque regions, consistent with improved local efferocytic clearance.

### Human plaque and systems-genetic analyses link mertk-related clearance to plaque burden

Independent coronary plaque data provided complementary disease-state evidence. In GSE184073, the plotted cell-level distribution of the Mertk homolog in macrophages was higher in chronic-coronary-syndrome/stable-angina-associated than in acute-coronary-syndrome-associated coronary plaque cells (Supplementary Fig. 6 A, B). The source study pooled plaque CD45-positive cells from four chronic-coronary-syndrome/stable-angina patients and three acute-coronary-syndrome patients within each clinical group before single-cell RNA sequencing, so donor-specific expression was not available. This comparison was therefore treated as descriptive cell-level evidence and not as a donor-level test of histological plaque stability. Nevertheless, the higher cell-level Mertk expression in chronic-coronary-syndrome/stable-angina-associated plaques supports a potential association with a clinically more stable, non-acute plaque phenotype. Across FUSION transcriptome-wide association framework, FOCUS, and SuSiE/FINEMAP analyses of CPB-associated signals, prioritized gene sets were enriched for receptor-mediated endocytosis, regulation of endocytosis, phagocytic vesicles, scavenger-receptor activity, and apoptotic-cell clearance (Supplementary Fig. 6C–E). The underlying analyses yielded 61 transcriptome-wide significant gene-panel associations, 120 FOCUS credible-set gene-panel entries with posterior inclusion probability greater than 0.90, and 355 variant records supported by at least one of SuSiE or FINEMAP (Supplementary Tables 5–7).

Proteome-wide Mendelian randomization identified 13 plasma protein-exposure associations representing 12 unique genes that remained significant after Bonferroni correction (Supplementary Table 3). Among these, genetically proxied higher circulating GAS6, a Mertk ligand, was associated with lower CPB liability (OR = 0.94, 95% CI 0.91–0.97; P = 2.03 × 10⁻^5^; Bonferroni-adjusted P = 0.0354; Supplementary Fig. 6 F). These independent plaque-expression and systems-genetic results provided genetic support linking Mertk-related apoptotic-cell clearance to systemic plaque burden and motivated subsequent mechanistic testing.

### Bmal1 maintains mertk expression and promotes mertk promoter activity in macrophages

**Fig. 6:**
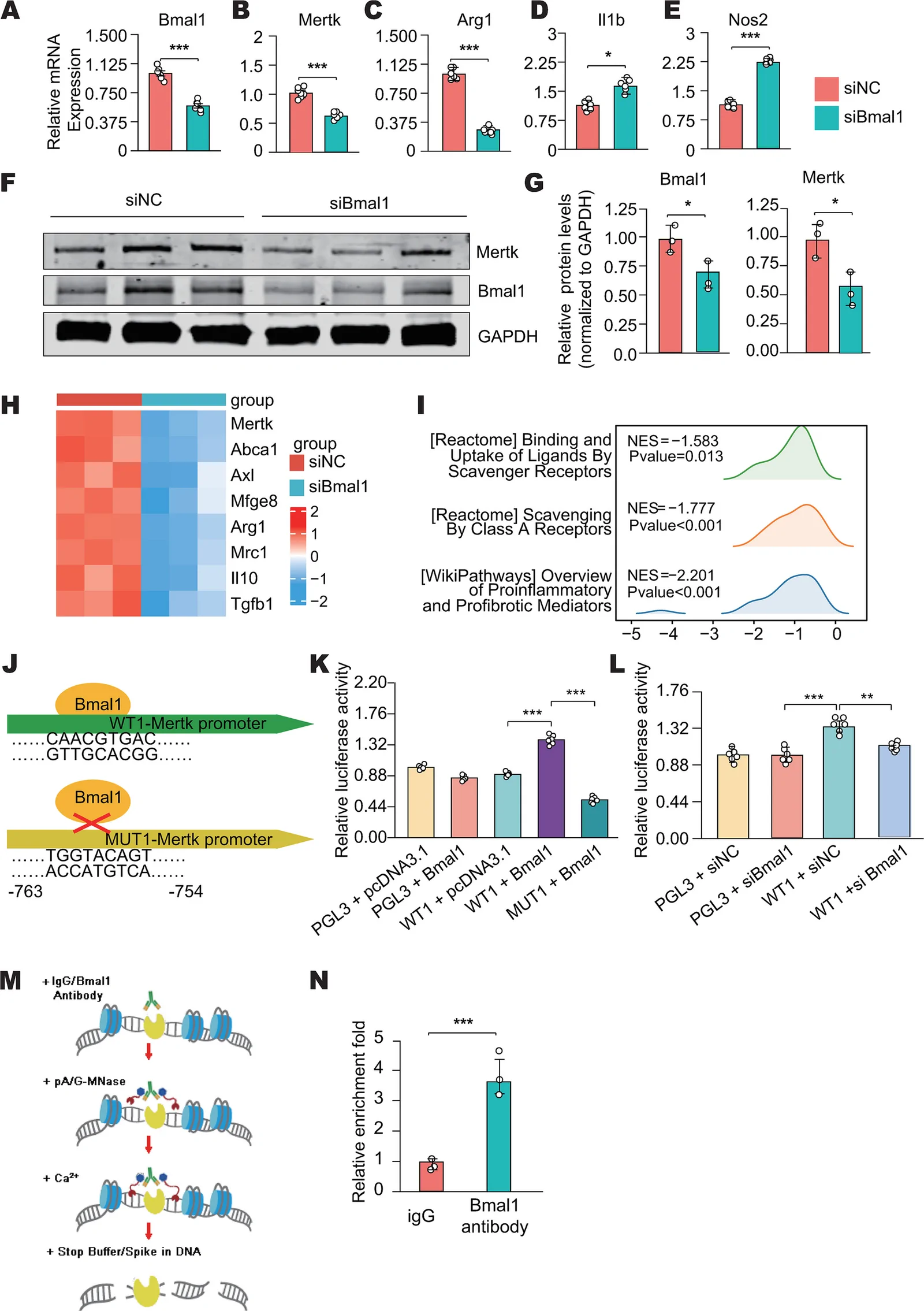
Bmal1 maintains Mertk expression and directly regulates the Mertk promoter in macrophages. **A**–**E** Relative Bmal1, Mertk, Arg1, Il1b, and Nos2 mRNA expression in mouse bone marrow-derived macrophages (BMDMs) transfected with negative-control siRNA (siNC) or Bmal1 siRNA (siBmal1; n = 6 independent experiments). **F**, **G** Immunoblotting and densitometric quantification of Bmal1 and Mertk protein abundance after Bmal1 knockdown; GAPDH was the loading control (n = 3 independent experiments). **H** Row-scaled heatmap of clearance- and repair-associated transcripts after Bmal1 knockdown (*n* = 3 independent experiments per group). **I** Gene set enrichment analysis showing altered scavenger-receptor and proinflammatory/profibrotic mediator programs; normalized enrichment scores (NES) and P values are displayed. **J** Schematic of the wild-type (WT1) and mutant (MUT1) Mertk promoter constructs for the potential Bmal1-binding site at − 763 to − 754 relative to the transcription start site. **K** Relative luciferase activity in HEK293T cells transfected with the indicated empty reporter, WT1, or MUT1 reporter and Bmal1 expression or matched pcDNA3.1 empty vector (*n* = 6 independent experiments). **L** Relative luciferase activity in BMDMs transfected with pGL3-Basic or WT1 reporter together with siNC or siBmal1 (n = 6 independent experiments). **M** Schematic of the cleavage under targets and release using nuclease (CUT&RUN) assay. **N** CUT&RUN-qPCR measurement of Bmal1 occupancy at the tested Mertk promoter region (*n* = 3 paired independent experiments). IgG was normalized to 1 for graphical presentation; statistical analysis was performed on ΔCt values. Data are mean ± SEM. A–G were analyzed by unpaired two-sided Student's t test, K and L by one-way ANOVA followed by Tukey's multiple-comparison test, and N by paired two-sided Student's t test. All reported experimental-comparison P values are two-sided. *P < 0.05; **P < 0.01; ***P < 0.001. The second predicted promoter site and its reporter results are shown in Supplementary Fig. 7

To test the relationship between Bmal1 and Mertk, Bmal1 was silenced in mouse bone marrow-derived macrophages. Bmal1 knockdown reduced Bmal1, Mertk, and *Arg1* mRNA expression while increasing *Il1b* and *Nos2* expression (Fig. 6A–E). Immunoblotting confirmed lower Bmal1 and Mertk protein abundance in siBmal1-treated cells (Fig. 6F, G). These results linked Bmal1 abundance to Mertk expression and the macrophage transcriptional state.

Transcriptome profiling provided broader support for this relationship. Clearance- and repair-associated genes, including Mertk, *Abca1*, *Axl*, *Mfge8*, *Arg1*, *Mrc1*, *Il10*, and *Tgfb1*, were lower after Bmal1 knockdown (Fig. 6H). Gene set enrichment analysis identified significant changes in ligand binding and uptake by scavenger receptors, scavenging by class A receptors, and proinflammatory/profibrotic mediator programs (Fig. 6I). Together with the qPCR results, these data show that Bmal1 loss remodeled macrophage receptor and inflammatory transcriptional programs.

Promoter-sequence analysis identified two potential Bmal1-binding sites at − 763 to − 754 and − 111 to − 101 relative to the Mertk transcription start site (Fig. 6J). In HEK293T cells, Bmal1 overexpression increased activity of the WT1 Mertk promoter reporter, whereas mutation of the first site reduced Bmal1-responsive reporter activity (Fig. 6K). The second predicted site and its matched mutant were evaluated separately (Supplementary Fig. 7 A); neither WT2 nor MUT2 showed detectable Bmal1-responsive reporter activity (Supplementary Fig. 7B). These results supported site-selective regulation and prioritized the first promoter region for subsequent macrophage reporter and occupancy analyses.

The regulatory effect was reproduced in macrophages: Bmal1 knockdown reduced WT1 Mertk promoter activity in bone marrow-derived macrophages (Fig. 6L). CUT&RUN-qPCR showed greater enrichment of the tested Mertk promoter region in anti-Bmal1 samples than in IgG controls (Fig. 6M, N), supporting Bmal1 occupancy at this region. Together, promoter mutagenesis, macrophage reporter analysis, and chromatin occupancy support direct transcriptional regulation of Mertk by Bmal1.

### Mertk mediates Bmal1-dependent preservation of macrophage efferocytosis under lipid stress

**Fig. 7:**
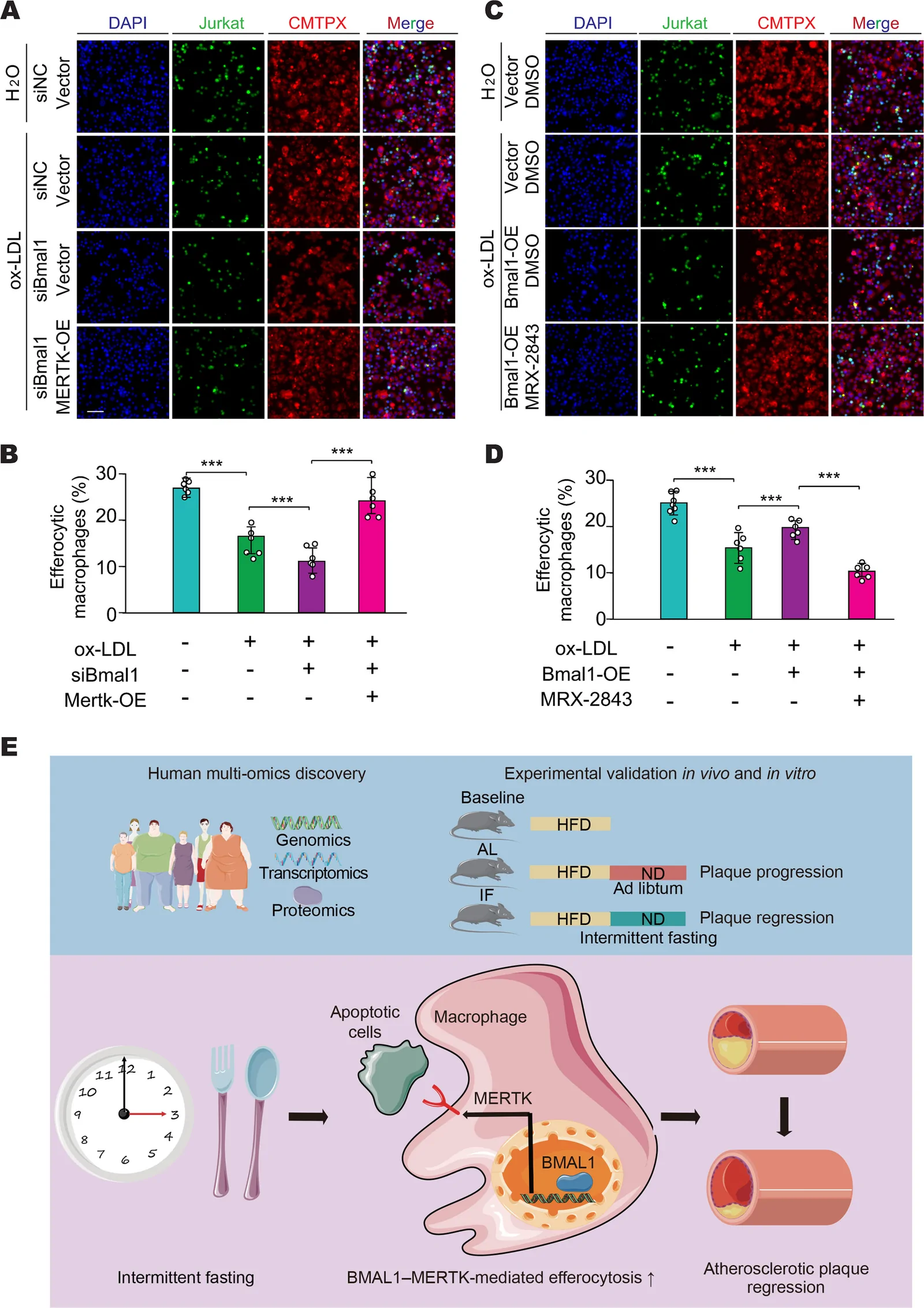
Mertk mediates Bmal1-dependent preservation of macrophage efferocytosis under lipid stress and supports a proposed working model of plaque remodeling. **A**, **B** Representative images (**A**) and quantification (**B**) of efferocytosis in BMDMs under untreated control conditions, oxidized low-density lipoprotein (ox-LDL) exposure, ox-LDL plus Bmal1 knockdown, or ox-LDL plus Bmal1 knockdown with Mertk re-expression. **C**, **D** Complementary gain-of-function and inhibitor experiment under untreated control conditions, ox-LDL exposure, ox-LDL plus Bmal1 overexpression, or ox-LDL plus Bmal1 overexpression with the Mertk/FLT3 kinase inhibitor MRX-2843 (150 nM; 2-h pretreatment). Apoptotic Jurkat targets were labeled with CFDA-SE (green), BMDMs with CMTPX (red), and nuclei with DAPI (blue). Scale bar, 50 μm; the bar shown at the lower left of A applies to both A and C. The efferocytosis index was the percentage of red macrophages containing at least one internalized green apoptotic target. For each condition, BMDMs isolated separately from three donor mice were maintained in three donor-specific culture dishes, with each dish constituting one biological replicate (*n* = 3). Two completely nonoverlapping fields were acquired from each dish and retained as separate field-level measurements; therefore, six points are shown per condition (two fields nested within each of three biological replicates). Data are mean ± SEM and were analyzed by one-way ANOVA followed by Tukey's multiple-comparison test. All reported experimental-comparison P values are two-sided. ***P < 0.001. **E** Proposed working model integrating human systems-genetic candidate prioritization with the mouse plaque-remodeling phenotype and macrophage experiments. The functional Bmal1–Mertk dependency was established in cultured macrophages; its contribution to the complete in vivo response to IF remains a proposed link that requires macrophage-specific genetic validation. CFDA-SE, carboxyfluorescein diacetate succinimidyl ester; CMTPX, CellTracker Red CMTPX

We next tested whether Mertk functionally mediated the effect of Bmal1 on macrophage efferocytosis. CFDA-SE-labeled apoptotic Jurkat cells were co-cultured with CMTPX-labeled bone marrow-derived macrophages, and efferocytosis was quantified as the proportion of macrophages containing internalized apoptotic targets. Oxidized low-density lipoprotein reduced efferocytic activity relative to the untreated control, and Bmal1 knockdown further impaired efferocytosis. Re-expression of Mertk in Bmal1-deficient macrophages restored efferocytic activity toward the control level (Fig. 7A, B).

A complementary gain-of-function and inhibitor experiment tested the same pathway in the opposite direction. Bmal1 overexpression partially restored efferocytosis in oxidized low-density lipoprotein-treated macrophages, whereas Mertk inhibition with MRX-2843 abolished this improvement and reduced efferocytic activity (Fig. 7C, D). The concordant genetic rescue and pharmacological inhibition results identify Mertk activity as a functional mediator of the pro-efferocytic effect of Bmal1 in this cellular setting.

Together, the in vivo plaque-remodeling phenotype, multi-omic prioritization, promoter-level regulation, and macrophage rescue experiments support a model in which intermittent fasting engages a Bmal1–Mertk program that enhances apoptotic-cell clearance and contributes to regression and favorable remodeling of established plaques (Fig. 7E).

## Discussion

Integrating population multi-omics with animal and cellular experiments, this study shows that intermittent fasting was associated with remodeling of established atherosclerotic plaques and identifies macrophage Bmal1-Mertk signaling as a contributing mechanism. In mice, intermittent fasting reduced plaque size relative to both the ad libitum-fed and week-14 Baseline groups and improved histological features of plaque stability, despite comparable endpoint lipid profiles in the two intervention groups. Aortic transcriptomic analyses prioritized Bmal1, whereas human single-cell, plaque-expression, and systems-genetic analyses prioritized Mertk and supported macrophage involvement. CUT&RUN supported Bmal1 occupancy at the examined Mertk promoter region, promoter-reporter assays supported Bmal1-dependent Mertk promoter activation, and loss-of-function and rescue experiments showed that Mertk contributes to Bmal1-associated macrophage efferocytosis. Together, these findings connect a defined intermittent-fasting regimen to local plaque remodeling while avoiding the inference that the Bmal1-Mertk axis is the sole mediator.

The three-group design distinguishes plaque status at intervention onset from subsequent changes after the switch to normal diet. The week-14 Baseline group provides a cross-sectional reference for established lesions, whereas the ad libitum-fed group indicates how plaques behaved after the switch to normal diet without fasting. Lesion burden was higher in ad libitum-fed mice than at Baseline but lower in intermittent-fasting mice than in both groups. At the group level, this pattern is consistent with net plaque regression relative to the week-14 reference, although the cross-sectional design cannot completely exclude a contribution from suppression of ongoing progression. Baseline-sacrifice designs have been used to infer regression in experimental atherosclerosis [75, 76]. The 14-week induction period was selected to generate substantial, histologically established lesions while retaining a window in which plaque remodeling could be detected during the subsequent 4-week intervention; it was not selected on the assumption that lesion burden had reached a stable plateau. Baseline histology demonstrated lipid-rich plaques with necrotic-core, fibrous-cap, and collagen features, confirming established lesion architecture but not temporal stabilization. A longer induction interval, such as 18 weeks, would provide a complementary test in more advanced disease; however, lesion stage itself can alter regression capacity, and a longer induction period alone would not demonstrate that a stable plateau had been reached [31, 78]. Future studies incorporating multiple pre-intervention sacrifice points, serial vascular imaging, and parallel 14- and 18-week induction cohorts will be required to distinguish within-animal regression from suppression of ongoing progression more definitively. Established lesions can continue to remodel after lipid lowering through changes in monocyte recruitment, inflammatory resolution, cell death, and matrix turnover [31, 77, 78]. The smaller lesions and more favorable stability features observed during intermittent fasting [79–81] therefore support local tissue remodeling beyond differences captured by endpoint circulating lipid concentrations. Consistent with this stage-dependent response, prior work in Ldlr⁻/⁻ mice showed that lesion size and macrophage area could initially increase after withdrawal of a Western-type diet despite a marked reduction in plasma cholesterol[78]. In the present study, the higher plaque CCL2 signal in ad libitum-fed mice than at Baseline supports persistence of a monocyte-recruiting inflammatory signal after the diet switch, whereas intermittent fasting suppressed CCL2. However, CCL2 immunofluorescence is a chemokine readout rather than a direct cell-tracking measurement of monocyte influx.

The observed response should be interpreted as specific to the intermittent-fasting schedule used here, because fasting interventions differ in duration, timing, feeding phase, and biological context [82]. Host metabolic state can also modify fasting-associated immunometabolic responses [83]. In this experiment, similar average daily food intake between ad libitum-fed and intermittent-fasting mice did not exclude effects of body-weight change or temporal redistribution of energy intake [84]. The accompanying increase in the anti-inflammatory cytokine IL10, together with lower F4/80, MMP9, and CCL2 plaque signals, supports a less inflammatory macrophage-associated plaque phenotype [21, 85]. These marker changes may reflect an integrated response to fasting-feeding transitions, energy balance, and circadian entrainment. These data support a regimen-specific vascular effect and should not be generalized to all intermittent-fasting schedules.

Mechanistic evidence converged on a macrophage Bmal1-Mertk-efferocytosis axis. Bmal1 has established roles in peripheral metabolic and immune regulation [86, 87] and in macrophage inflammatory programs [88–90]. In this study, Bmal1 knockdown reduced Mertk expression and efferocytosis, whereas Mertk re-expression restored efferocytic activity. CUT&RUN supported Bmal1 occupancy at the examined predicted region of the Mertk promoter, and reporter assays supported Bmal1-dependent promoter activation; these experiments do not by themselves establish that the predicted motif is required in vivo. Prior studies show that Mertk dysfunction or cleavage impairs efferocytosis and promotes apoptotic-cell accumulation and plaque necrosis [91–93], whereas effective efferocytosis supports inflammatory resolution [94]. Concordant with the human macrophage-state patterns and cultured-cell experiments, intermittent fasting increased the plaque ratio of Mac-2-positive macrophage-associated to free apoptotic cells. Because this measure incorporates the spatial relationship between macrophages and apoptotic targets, it provides tissue-level support for enhanced efferocytic engagement, whereas the cultured macrophage experiments provide the direct functional evidence. The combined data therefore place Mertk as a functional downstream contributor to Bmal1-associated macrophage efferocytosis. Bmal1 was prioritized over the concurrently upregulated Npas2 because its established macrophage-intrinsic functions provided a direct route to test the macrophage/efferocytosis hypothesis; the present study does not exclude a contribution from Npas2.

Population multi-omics and public human single-cell analyses provide human relevance and candidate prioritization rather than direct evidence of an intermittent-fasting effect in people [95, 96]. CPB represents shared inherited liability across several arterial phenotypes; it is not an individual-level plaque measure, and its genetic associations cannot establish the effect of the mouse intervention. Gene-level association, transcriptome-wide association, proteome-wide Mendelian randomization, and fine-mapping converged on genes involved in apoptotic-cell recognition, endocytosis, and clearance, while cell-resolved analyses linked the human homologs of Bmal1 and Mertk to macrophage states in human plaques. These data strengthen the rationale for experimental testing, but the observational single-cell patterns and computational perturbations do not prove that Bmal1-Mertk signaling mediates fasting-associated plaque remodeling in humans.

From a translational perspective, the results suggest two testable directions rather than an immediate therapeutic recommendation. ROR agonism and the clock-amplitude modulator nobiletin can alter circadian programs [97, 98], and nobiletin-associated vascular effects have also been linked to PPARG/CD36-dependent lipid handling [99]. Plaque- or macrophage-targeted Mertk delivery has shown preclinical activity in nanoparticle studies [100]. However, because Mertk also participates in immune and platelet biology [25, 100, 101], future work should prioritize cell-selective delivery, safety, dosing, and timing. These observations make diet-independent modulation biologically plausible, but no pharmacological strategy has yet been shown to reproduce the complete plaque-remodeling effect of intermittent fasting through selective BMAL1-MERTK activation. Clinical translation will also require testing whether feasible human intermittent-fasting schedules reproduce the vascular and immune effects observed here.

Several limitations bound the interpretation of the animal and mechanistic findings. First, the Baseline group was cross-sectional, and neither serial vascular imaging nor additional pre-intervention time points were obtained. The lower lesion burden in intermittent-fasting mice is therefore consistent with group-level regression but does not demonstrate within-animal regression or establish that week-14 lesions had reached a stable plateau. Second, pair-fed or weight-matched controls and direct stress measurements were not included. Similar average daily food intake cannot fully separate fasting timing from energy distribution, weight change, metabolic adaptation, or stress. Third, only male mice were studied, limiting generalizability because sex may influence lipid metabolism, atherosclerosis progression, immune responses, and peripheral circadian rhythms. Future studies should replicate these findings in female mice and test for sex-by-intervention interactions. The animals were maintained on a 12 h light/12 h dark cycle, with food withdrawal and refeeding occurring at 20:00 and each 24 h fast spanning a complete light–dark cycle. Nevertheless, the circadian phase of food withdrawal and refeeding may influence Bmal1 expression, metabolic switching, and immune responses. Thus, the observed effects may reflect both fasting duration and the phase of the fasting-refeeding cycle. Future phase-controlled studies will be required to determine whether changing fasting onset modifies the vascular response. Fourth, the mechanistic experiments focused on macrophages, although T cells, B cells, vascular smooth-muscle cells, and endothelial cells may also contribute [102]. Macrophage-specific Bmal1 or Mertk loss-of-function was not tested during intermittent fasting in vivo, so the Bmal1-Mertk axis should be interpreted as contributory rather than necessary. Finally, the plaque efferocytosis index was derived from static spatial overlap, contact, or encirclement between TUNEL-positive apoptotic cells and Mac-2-positive macrophages. This morphology-based measure cannot distinguish completed internalization from surface adhesion or close apposition and should therefore be interpreted as a histologic surrogate of efferocytic engagement rather than a direct live-cell measure of engulfment. Histological sampling also cannot capture all spatial heterogeneity within established plaques, even when sections and fields are averaged within each mouse.

The human and computational components impose additional limits. The genetic analyses were derived predominantly from European-ancestry cohorts, restricting transferability across populations. Public single-cell datasets are observational and remain sensitive to sampling, dropout, batch correction, cell annotation, and trajectory-root selection; pseudotime therefore represents an ordered cell-state manifold rather than observed temporal progression. Symptomatic and asymptomatic carotid plaque groups are not direct surrogates for histologically unstable and stable plaques, and transcript-level associations require independent protein-level confirmation. Likewise, the pooled chronic-coronary-syndrome/stable-angina and acute-coronary-syndrome plaque samples in GSE184073 do not support donor-level inference. The mouse intermittent-fasting regimen should not be equated with common human intermittent-fasting or time-restricted-eating schedules. Replication in diverse populations, independent plaque-tissue validation, in vivo cell-specific perturbation, and prospective studies using clinically feasible schedules will be required to establish generalizability and translational value. Virtual-knockout rankings likewise depend on the inferred network, preprocessing, and model settings. Cross-dataset concordance provides a robustness check, but formal sensitivity analyses using alternative clustering, batch-correction, or network-inference choices were not performed.

## Supplementary Information

Additional file 1: Fig. S1 Systemic variables were comparable at the week-14 group-allocation point.Serum total cholesterol, triglycerides, low-density lipoprotein cholesterol, high-density lipoprotein cholesterol, and non-esterified fatty acids, respectively, after 14 weeks of high-fat, high-cholesterol diet and before the group-specific intervention.Body weight at the same time point. Group labels indicate subsequent allocation to the week-14 Baseline, ad libitum-fed, or intermittent-fastinggroups; no group had yet received the assigned intervention. n = 8 mice per group, and each point represents one mouse. Data are mean ± SEM and were analyzed by one-way ANOVA followed by Tukey's multiple-comparison test. All reported animal-comparison P values are two-sided. ns, not significant. Fig. S2 IF reduces plaque MMP9 and CCL2 signals.Representative MMP9 immunofluorescence images of aortic-root plaquesand relative plaque-region mean fluorescence intensityin the week-14 Baseline, ad libitum-fed, and intermittent-fastinggroups.Representative CCL2 immunofluorescence imagesand relative plaque-region MFI. MMP9 and CCL2 are shown in green and nuclei in blue. Red boxes identify the enlarged plaque regions. Scale bars, 500 μm in overview images and 300 μm in enlarged images. Quantification was restricted to the prespecified aortic-root plaque region; outer arterial-wall and elastic-lamina signals were excluded. n = 8 mice per group, and each point represents one mouse. Data are mean ± SEM and were analyzed by one-way ANOVA followed by Tukey's multiple-comparison test. All reported animal-comparison P values are two-sided. **P < 0.01; ***P < 0.001. Fig. S3 IF increases Bmal1 signal in CD68-positive plaque regions.Representative Bmal1 and CD68 immunofluorescence images of aortic-root plaques from the week-14 Baseline, ad libitum-fed, and intermittent-fastinggroups. Bmal1 is shown in red, CD68 in green, and nuclei in blue; yellow signal in merged images indicates spatial overlap. Red boxes identify the enlarged plaque regions. Scale bars, 500 μm in overview images and 300 μm in enlarged images.Relative Bmal1 MFI within CD68-positive plaque regions. n = 8 mice per group, and each point represents one mouse. Data are mean ± SEM and were analyzed by one-way ANOVA followed by Tukey's multiple-comparison test. All reported animal-comparison P values are two-sided. **P < 0.01; ***P < 0.001. Fig. S4 IF promotes an IL10-associated anti-inflammatory phenotype in aortic-root plaques.Representative co-immunofluorescence images of the anti-inflammatory cytokine IL10 and the macrophage marker F4/80 in aortic-root plaques from the week-14 Baseline, ad libitum-fed, and intermittent-fastinggroups. IL10 is shown in green, F4/80 in red, and nuclei in blue. Red boxes identify the enlarged plaque regions. Scale bars, 500 μm in overview images and 300 μm in enlarged images.Relative plaque-region mean fluorescence intensityof IL10and F4/80, quantified separately within the same prespecified plaque region of interest. n = 8 mice per group, and each point represents one mouse. Data are mean ± SEM and were analyzed by one-way ANOVA followed by Tukey's multiple-comparison test. All reported animal-comparison P values are two-sided. **P < 0.01; ***P < 0.001. Fig. S5 Bmal1 and Mertk homolog expression is correlated in human whole blood. Scatter plot of log2-transformed transcripts-per-million expression values for the Bmal1 and Mertk homologs in GTEx whole-blood samples obtained through GEPIA. The analysis included n = 337 samples, and each point represents one sample. The Spearman P value is two-sided. Spearman correlation: ρ = 0.66, P = 4.9 × 10⁻⁴⁴. Fig. S6. Human plaque and systems-genetic analyses connect Mertk-related clearance pathways with CPB.Uniform manifold approximation and projectionof T cells, macrophages, B cells, and natural killercells from chronic coronary syndrome/stable angina pectoris-associated and acute coronary syndrome-associated human coronary plaques in GSE184073, displayed in the panel as stable and unstable, respectively. The source data pooled CD45-positive plaque cells from four CCS/SAP patients without high-intensity plaque signals on non-contrast T1-weighted magnetic resonance imaging and three ACS patients within each clinical group before single-cell RNA sequencing.Cell-level distribution of normalized Mertk homolog expression in macrophages from the two clinical groups. The plotted comparison used a two-sided Wilcoxon rank-sum test; ***P < 0.001. Because donor identity was not retained after pooling, this panel is presented as a descriptive cell-level association rather than donor-level or histological plaque-stability inference.Functional enrichment of genes prioritized from circulatory plaque burdensummary statistics by FUSION transcriptome-wide association analysis with Bonferroni correction, FOCUS credible-set analysis, and shared SuSiE/FINEMAP credible sets. Bubble size indicates gene count and color indicates P value. Complete gene- and variant-level outputs are provided in Supplementary Tables 5–7.Forest plot of circulating-protein associations with CPB that remained significant after analysis-specific Bonferroni correction in proteome-wide Mendelian randomization using deCODE pQTL instruments. Squares indicate odds ratios and horizontal lines indicate 95% confidence intervals; the vertical line denotes an odds ratio of 1. GAS6, a Mertk ligand, is highlighted in red. Complete results are provided in Supplementary Table 3. Fig. 7 The second predicted Mertk promoter site shows no detectable Bmal1-responsive activity.Schematic of the wild-typeand matched mutantMertk promoter constructs spanning the second predicted Bmal1-binding site at −111 to −101 relative to the transcription start site.Relative luciferase activity in HEK293T cells transfected with pGL3-Basic, WT2, or MUT2 reporter together with Bmal1 expression or matched pcDNA3.1 empty vector, as indicated. Data are mean ± SEM and were analyzed by one-way ANOVA followed by Tukey's multiple-comparison test. All reported experimental-comparison P values are two-sided. ns, not significant

Additional file 2: Table S1: GWAS summary data sources for structural equation modeling

Additional file 3: Table S2: GenomicSEM model parameters for the Circulatory Plaque Burden latent variable

Additional file 4: Table S3: Genetically supported directional associations of plasma proteins with Circulatory Plaque Burdenidentified by proteome-wide Mendelian randomization

Additional file 5: Table S4: Gene-level associations with Circulatory Plaque Burdenidentified by MAGMA

Additional file 6: Table S5: Transcriptome-wide associations with Circulatory Plaque Burdenidentified using FUSION

Additional file 7: Table S6: FOCUS credible sets of candidate genes for Circulatory Plaque Burden

Additional file8: Table S7: Variant-level credible sets for Circulatory Plaque Burdenidentified by SuSiE and FINEMAP

Additional file 9: Table S8: Primer sequences used for quantitative real-time PCRanalysis

## Data Availability

All data supporting the findings of this study are available within the paper and its additional files. The original transcriptome sequencing datasets generated during the current study have not been deposited into a public repository but are available from the corresponding authors upon reasonable request. Publicly available datasets reanalyzed in this study (GSE253903, GSE314596) are accessible via the NCBI GEO database at https://www.ncbi.nlm.nih.gov/geo/query/acc.cgi?acc=GSE253903 and https://www.ncbi.nlm.nih.gov/geo/query/acc.cgi?acc=GSE314596. GTEx data were obtained via the GTEx portal (https://gtexportal.org/). deCODE plasma proteomics data are available as described in the original publication (Ferkingstad et al., Nature Genetics, 2021).

## Abbreviations

AA: Abdominal aortic phenotype
AC: Apoptotic cell
AL: Ad libitum feeding
ACS: Acute coronary syndrome
ASCVD: Atherosclerotic cardiovascular disease
BMDM: Bone marrow-derived macrophage
CAD: Coronary artery disease
CCS: Chronic coronary syndrome
CFDA-SE: Carboxyfluorescein diacetate succinimidyl ester
CI: Confidence interval
CMTPX: CellTracker Red CMTPX
CPB: Circulatory plaque burden
CUT&RUN: Cleavage under targets and release using nuclease
DAPI: 4′,6-Diamidino-2-phenylindole
DC: Dendritic cell
DEG: Differentially expressed gene
DMEM: Dulbecco's modified Eagle medium
EVG: Elastica van Gieson
FBS: Fetal bovine serum
FC: Fibrous cap
FDR: False discovery rate
FINEMAP: Bayesian genetic fine-mapping software
FOCUS: Fine-mapping of causal gene sets
FUMA: Functional Mapping and Annotation
FUSION: Functional summary-based imputation framework
GSEA: Gene set enrichment analysis
GTEx: Genotype-Tissue Expression
GWAS: Genome-wide association study
GWAS-SEM: Genome-wide association study structural equation modeling
HDL: High-definition likelihood
HDL-C: High-density lipoprotein cholesterol
HE: Hematoxylin and eosin
HFD: High-fat, high-cholesterol diet
IF: Intermittent fasting
LDSC: Linkage disequilibrium score regression
LDL-C: Low-density lipoprotein cholesterol
MAGMA: Multi-marker Analysis of GenoMic Annotation
M-CSF: Macrophage colony-stimulating factor
MFI: Mean fluorescence intensity
Mφ: Macrophage
MUT: Mutant
ND: Normal diet
NC: Necrotic core
NEFA: Non-esterified fatty acids
NES: Normalized enrichment score
NK: Natural killer cell
OR: Odds ratio
ox-LDL: Oxidized low-density lipoprotein
PAD: Peripheral artery disease
PBS: Phosphate-buffered saline
PLACO: Pleiotropic analysis under a composite null hypothesis
pQTL: Protein quantitative trait locus
PW-MR: Proteome-wide Mendelian randomization
qPCR: Quantitative polymerase chain reaction
ROR: Retinoic acid receptor-related orphan receptor
SAP: Stable angina pectoris
scRNA-seq: Single-cell RNA sequencing
SEM: Standard error of the mean
siRNA: Small interfering RNA
SMC: Smooth muscle cell
SNP: Single-nucleotide polymorphism
SuSiE: Sum of Single Effects
TC: Total cholesterol
TG: Triglycerides
TPM: Transcripts per million
TUNEL: Terminal deoxynucleotidyl transferase dUTP nick-end labeling
TWAS: Transcriptome-wide association study
UMAP: Uniform manifold approximation and projection
WT: Wild type
